# Evolution of Daily Gene Co-expression Patterns from Algae to Plants

**DOI:** 10.3389/fpls.2017.01217

**Published:** 2017-07-13

**Authors:** Pedro de los Reyes, Francisco J. Romero-Campero, M. Teresa Ruiz, José M. Romero, Federico Valverde

**Affiliations:** ^1^Plant Development Unit, Institute for Plant Biochemistry and Photosynthesis, Consejo Superior de Investigaciones Científicas, Universidad de Sevilla Seville, Spain; ^2^Department of Computer Science and Artificial Intelligence, Universidad de Sevilla Seville, Spain

**Keywords:** daily rhythmic genes, evolution, co-expression networks, systems biology, circadian, *Arabidopsis*, *Chlamydomonas*, *Ostreococcus*

## Abstract

Daily rhythms play a key role in transcriptome regulation in plants and microalgae orchestrating responses that, among other processes, anticipate light transitions that are essential for their metabolism and development. The recent accumulation of genome-wide transcriptomic data generated under alternating light:dark periods from plants and microalgae has made possible integrative and comparative analysis that could contribute to shed light on the evolution of daily rhythms in the green lineage. In this work, RNA-seq and microarray data generated over 24 h periods in different light regimes from the eudicot *Arabidopsis thaliana* and the microalgae *Chlamydomonas reinhardtii* and *Ostreococcus tauri* have been integrated and analyzed using gene co-expression networks. This analysis revealed a reduction in the size of the daily rhythmic transcriptome from around 90% in *Ostreococcus*, being heavily influenced by light transitions, to around 40% in *Arabidopsis*, where a certain independence from light transitions can be observed. A novel Multiple Bidirectional Best Hit (MBBH) algorithm was applied to associate single genes with a family of potential orthologues from evolutionary distant species. Gene duplication, amplification and divergence of rhythmic expression profiles seems to have played a central role in the evolution of gene families in the green lineage such as *Pseudo Response Regulators* (*PRRs*), *CONSTANS-Likes* (*COLs*), and *DNA-binding with One Finger* (*DOFs*). Gene clustering and functional enrichment have been used to identify groups of genes with similar rhythmic gene expression patterns. The comparison of gene clusters between species based on potential orthologous relationships has unveiled a low to moderate level of conservation of daily rhythmic expression patterns. However, a strikingly high conservation was found for the gene clusters exhibiting their highest and/or lowest expression value during the light transitions.

## Introduction

The evolution of the green lineage of photosynthetic organisms from unicellular green algae to land plants is subject to intense research. Recently, genomic analysis have been applied to study key events in the evolution of the green lineage such as terrestrialization (Delwiche and Cooper, 2015; de Vries et al., 2016). Nevertheless, the integration of genomics and transcriptomics to study the evolution of gene expression patterns in the green lineage has only recently been explored (Romero-Campero et al., 2013; Ruprecht et al., 2017). Photosynthetic organisms are particularly influenced by daily light/dark transitions as their main energy income is a very demanding light-dependent process. Therefore, those plant ancestors (whose extant representative species are unicellular green algae) that were able to anticipate daily fluctuations and schedule their physiological processes accordingly, evolved into the present plant species (Mora-García et al., 2017). Transcriptomics have become a powerful tool to study the global influence of daily light/dark cycles in photosynthetic organisms. Thus, in higher plants, it has been described that between a third and a half of their gene expression is regulated by the circadian clock (Covington et al., 2008; Michael et al., 2008), whereas in algae between 80 and 90% of the transcriptome follows light-dependent daily rhythmic patterns (Monnier et al., 2010; Zones et al., 2015). It seems then that daily rhythmic regulation of gene expression reached a maximum in algae and has substantially decreased in Spermatophytes. Key biological processes such as starch and sugar metabolism exhibit daily rhythmic patterns in both plants and algae (Bläsing et al., 2005; Sorokina et al., 2011), but these analysis remain fragmented and focused on individual species. In order to better understand the evolution of daily rhythmic gene expression between algae and plants, confirm the tendency to decrease daily regulation and determine the evolution of daily regulated biological processes from algae to plants, a series of comparative Systems Biology analysis integrating genomics and transcriptomics has been used in this study.

The availability of massive amounts of transcriptomic data obtained from different species under equivalent environmental conditions has enabled the use of comparative transcriptomics methodologies to study the evolution of key biological processes (Trachana et al., 2010). In this work, database available RNA-seq and microarray data generated over 24 h periods in neutral days (ND: 12 h of light/12 h of dark) and long days (LD: 16 h of light/8 h of dark) conditions from three different model photosynthetic species (*Arabidopsis thaliana, Chlamydomonas reinhardtii*, and *Ostreococcus tauri*) have been integrated and analyzed. These photosynthetic eukaryotes represent distant phylogenetic groups. Among the *Chlorophyta* algae division, the marine Prasinophyceae microalga *Ostreococcus* is considered a representative of ancient microalgae, one of the smallest eukaryote (picoeukaryote) and constitutes an important part of sea phytoplankton (Derelle et al., 2006; Palenik et al., 2007). Due to its small genome (13 Mb), whose sequencing has recently been improved (Blanc-Mathieu et al., 2014), its non-flagellar small body (0.8 μm), its single copy organella (Henderson et al., 2007) and its planktonic life style, it has been considered as the smallest possible photosynthetic eukaryote (Raven et al., 2013) and promising organism for Systems Biology (Thommen et al., 2015). *Chlamydomonas* is a Chlorophyceae microalga and has been used as a model for photosynthetic organisms' studies for many years (Harris, 2001). *Chlamydomonas* has two polar flagella (10 μm body), a much bigger genome than *Ostreococcus* (120 Mb; Merchant et al., 2007) and lives in sweet water environments. Omics are currently intensely used to explore *Chlamydomonas* potential in biotechnological applications (Aucoin et al., 2016). As a representative Spermatophyte or seed plant, *Arabidopsis* has several characteristics that make it especially useful for Systems Biology studies in general and circadian experiments in particular, as well as a bigger genome and a complex physiology in comparison to microalgae (Van Norman and Benfey, 2009; Koornneef and Meinke, 2010).

*Arabidopsis* has been extensively used as a model to describe the basic aspects of plant circadian clock regulation over daily rhythms (Millar, 2016; Nohales and Kay, 2016). In a few words, it is formed by three interlocked positive/negative feedback loops. CIRCADIAN CLOCK-ASSOCIATED 1 (CCA1) and LATE ELONGATED HYPOCOTYL (LHY) myb transcription factors constitute the positive/negative “morning loop” together with PRR9, PRR7 and PRR5 (Nakamichi et al., 2012; Liu et al., 2013, 2016; Kamioka et al., 2016). The negative “central loop” is constituted by CCA1/LHY repression over the gene *TIME OF CAB EXPRESSION 1* (*TOC1*) that, in time, represses the “morning loop” genes and activates evening-regulated genes in a 24 h-long feed-back loop (Huang et al., 2012). Over this basic core, the circadian genes: *GIGANTEA* (*GI*), *ZEITLUPE* (*ZTL*), *EARLY LIGHT FLOWERING 3* (*ELF3*), *ELF4, LUX ARRHYTMO* (*LUX*) among others, constitute the “evening loop” that refines the clock and allows for the complex response to the changing daily conditions, including light and temperature inputs (Miyazaki et al., 2015). Outputs of this clock are cell wall synthesis, photosynthetic and starch metabolism genes, among many others (Adams and Carré, 2011). Taking *Arabidopsis* as model, a much simpler clock has been described in *Chlamydomonas* and *Ostreococcus*, where only some genes of the higher plant circadian clock have been identified so far (Mittag et al., 2005; Corellou et al., 2009). In this way, most evolutionary studies have focused on phylogenetic analyses of the key genes regulating the circadian clock (Serrano-Bueno et al., 2017) whereas the analysis of global rhythmic patterns conservation and evolution among different photosynthetic species still remains to be explored.

In this work, phylogenomic and transcriptomic data integration and analysis have been performed by gene co-expression networks construction (Romero-Campero et al., 2013; Gehan et al., 2015; Ruprecht et al., 2017) and a novel algorithm for the identification of potential orthologues called Multiple Best Bi-directional Hit (MBBH). Using clustering techniques, specific gene clusters or modules that showed a rhythmic daily regulation have been identified. Interestingly, these clusters consist of groups of highly co-expressed genes involved in particular biological processes such as cell cycle progression, photosynthesis and ribogenesis, revealing a significant temporal organization in their specific gene co-expression patterns. By comparing the gene modules identified in the different gene co-expression networks obtained for each species, it was possible to determine which biological processes have conserved a daily rhythmic co-expression pattern over the green lineage and which ones have evolved into different patterns. Additionally, a web based software tool, CircadiaNET (http://viridiplantae.ibvf.csic.es/circadiaNet/) has been developed that will allow researchers to independently analyze their circadian genes of interest studying the biological processes they are potentially involved in, the conservation or evolution of the gene co-expression patterns they follow, as well as the transcription factor (TF) binding sites that are significantly present in their promoters.

## Materials and methods

### Identification of putative orthologous proteins

**Figure 1:**
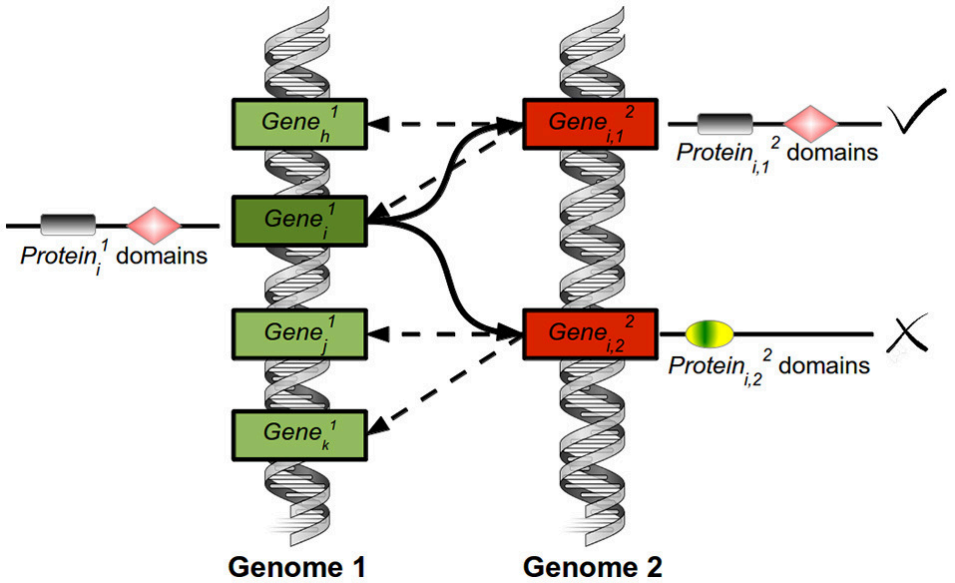
Explicative diagram of the Multiple Bidirectional Best Hits tool. For the ${Protein}_{i}^{\text{1}}$ encoded by the ${Gene}_{i}^{\text{1}}$ from the first species (deep green), the algorithm selects the genes (red) encoding the *N* proteins that exhibit the highest sequence similarity (${protein}_{i,\text{1}}^{\text{2}}$, ${protein}_{i,\text{2}}^{\text{2}}$*,…*${protein}_{i,n}^{\text{2}}$*)*. In this study, *N* was set to 20. These 20 proteins are called initial best hits. The same process is carried out for species 2. For each initial hit from species 2, the *N* genes (red) coding for proteins showing the highest sequence similarity are selected. Then, the initial best hits of the genes (green) coding for ${protein}_{i}^{\text{1}}$ from species 1 are filtered. Only those genes coding for proteins that exhibit ${protein}_{i}^{\text{1}}$ in their initial best hits are kept (marked by a √ symbol). Thus, bidirectionality is required. Additionally, the best bidirectional hits that do not share at least one domain with the corresponding protein are removed (marked by an X symbol).

The protein sequences of the three photosynthetic species analyzed in this study were downloaded from publicly available databases. *Chlamydomonas* v5.5 and *Arabidopsis* TAIR10 proteins were downloaded from Phytozome (http://www.phytozome.net/) (Goodstein et al., 2012), while *Ostreococcus* proteins v2 were downloaded from ORCAE (http://bioinformatics.psb.ugent.be/orcae/) (Sterck et al., 2012; Blanc-Mathieu et al., 2014). Using the tools available from the Pfam database (http://pfam.xfam.org/) (Punta et al., 2012), with default parameters (*E*-value 1.0), the protein domains in all protein sequences were identified. Potential homologous proteins between species were identified based on sequence similarity. We developed a variant of the Bidirectional Best Hit (BBH) that takes into account several candidates. We called this variant Multiple Bidirectional Best Hit (MBBH) and happen to be specially suited for duplication-enriched species such as *Arabidopsis*. The algorithm receives as input two fasta files containing the protein sequences of the two species to compare and the number *N* of multiple bidirectional best hits to consider (Figure 1). In this study *N* was fixed to 20. For each protein ${Prot}_{i}^{\text{1}}$ (encoded by ${Gene}_{i}^{\text{1}}$, deep green) from the first species, the *N* proteins (encoded by ${Gene}_{i,k}^{\text{2}}$, red) from the second species, ${Prot}_{i,\text{1}}^{\text{2}}$, ${Prot}_{i,\text{2}}^{\text{2}}$*,…,*${Prot}_{i,N}^{\text{2}}$, exhibiting the highest sequence similarity are selected using the Needleman-Wunsch algorithm implemented in the R Bioconductor package Biostrings (Pagès et al., 2016). These proteins will be called “initial best hits.” The same process is carried out for species 2: For each protein ${Prot}_{i}^{\text{2}}$ (encoded by ${Gene}_{i,k}^{\text{2}}$, red) from the second species, the *N* proteins from the first species, ${Prot}_{h}^{1}$, ${Prot}_{i}^{\text{1}}$*,…,*${Prot}_{N}^{\text{1}}$, exhibiting the highest sequence similarity are selected. Next, for each ${Prot}_{i}^{\text{1}}$ from the first species its initial best hits, ${Prot}_{i,\text{1}}^{\text{2}}$, ${Prot}_{i,2}^{\text{2}}$*,…,*${Prot}_{i,N}^{\text{2}}$, are filtered. Only those ${Prot}_{i,k}^{\text{2}}$ that present ${Prot}_{i}^{\text{1}}$ in their *N* initial best hits are kept. The same process is carried out for each protein ${Prot}_{j}^{\text{2}}$ from species 2 keeping only the initial best hits that present ${Prot}_{j}^{\text{2}}$ among their *N* initial best hits. An additional filtering process is performed by removing from the best bidirectional hits those that do not share at least one domain with the corresponding protein. Finally, we also assign putative orthology to proteins showing no MBBH target, but sharing exactly identical number and order of one or more, non-overlapping pfam domains. This means that due to the specificity of pfam domains, the presence of a single domain does not immediately imply the assignation of a putative orthologue as would be the case with specific higher plant domains such as NAM (**Figure 3C**) or specific microalgal domains. In this way, our approach combines global sequence similarity with protein domain structure and, additionally, allows for multiple best hits in order to capture the evolution of a single gene into a multi-gene family. In general, MBBH could be a useful tool for those researchers trying to identify orthologues from any given gene in different species, even when they belong to distantly related ones. The tools developed in this article will be operative in the web site: (http://viridiplantae.ibvf.csic.es/circadiaNet/).

### Transcriptomic data acquisition and processing

For the three photosynthetic species analyzed in this study, transcriptomic data comprising time series of 48 or 72 h were collected from the GEO database (https://www.ncbi.nlm.nih.gov/geo/) (Barrett et al., 2013). In the case of *Arabidopsis*, two different data sets were used. The first one identified with the accession number GSE3416 (Bläsing et al., 2005) consists of microarray data taken in ND conditions (12:12). The second one identified with the accession number GSE43865 (Rugnone et al., 2013) consists of RNA-seq data taken in LD conditions (16:8). For *Chlamydomonas*, a single RNA-seq data set was collected identified with the accession number GSE71469 (Zones et al., 2015) taken in ND conditions. For *Ostreococcus*, a single microarray data set was used, identified with the accession number GSE16422 (Monnier et al., 2010) taken in ND conditions. The microarray data were processed using the Robust Multi-array Average (RMA) implemented in the Bioconductor R package affy (Gautier et al., 2004). The RNA-seq data was processed following the Tuxedo protocol (Trapnell et al., 2012). Reads were mapped to the reference genomes downloaded from Phytozome using Tophat. Transcripts were assembled using Cufflinks. Default parameters were used for the different software tools. Gene expression levels were measured as FPKM (Fragments Per Kilobase of exon per Million fragments mapped). Since RNA-seq and microarray gene expression levels have different measurement units these data were normalized by subtracting the mean and dividing by the standard deviation using the R function scale from the base package. This allows representing both types of data on a common scale. Samples were collected every 3 or 4 h in the data analyzed in this study. In order to produce a smooth representation of the gene expression profiles in heatmap graphs these data were linearly interpolated to produce time series consisting of 24 h using the R function approx from the stats package. In heatmaps, genes were sorted according to their expression profile similarity using hierarchical clustering.

### Identification and clustering of rhythmic daily gene expression patterns

The detection of significant periodic patterns in the transcriptomic data analyzed was performed using RAIN (Rhythmicity Analysis Incorporating Nonparametric methods). This Bioconductor R package consists of functions that implement robust nonparametric methods for the detection of rhythms with arbitrary wave forms and pre-specified periods (Thaben and Westermark, 2014). The main function *rain* was used with the following parameters: A numeric matrix comprising the gene expression levels from the different biological replicates of the 24 h time series; a sampling interval of 4 h for *Arabidopsis* and 3 h for *Chlamydomonas* and *Ostreococcus*; a period of 24 h; and a number of three replicates for *Arabidopsis* and *Ostreococcus* and two for *Chlamydomonas*. A significance level α = 1% was chosen to assume that a certain gene exhibits a rhythmic daily expression pattern. The wave form of a gene exhibiting rhythmic daily expression was characterized using its peak (time point where the highest expression value is reached) and its trough (time point where the lowest expression value is reached). Daily rhythmic genes were thus classified into 16 different clusters according to their peaks and troughs at a particular time of the day, that is, all genes in the same cluster present their peaks and troughs at the same time point.

### Gene ontology term and pathway enrichment analysis

Gene Ontology (GO) terms associated to each *Arabidopsis* and *Chlamydomonas* gene were downloaded from Phytozome. For *Ostreococcus*, GO terms were downloaded from ORCAE. The Bioconductor R package topGO (Alexa and Rahnenfuhrer, 2016) was used to determine GO terms significantly enriched in different gene sets. The entire genome of the corresponding species was used as gene background. The statistical significance test selected was Fisher's exact test with a significance level α = 5%. The web based tool REVIGO (Supek et al., 2011) was used to remove redundancy from the enriched GO terms and produced a summary. The identification of enriched pathways in *Arabidopsis* gene sets was performed with the Bioconductor R package clusterProfiler (Yu et al., 2012). The enrichKEGG function was used with Bejamin-Hochberg as *p*-value correction method for the multiple testing and a *q*-value cutoff of 0.05.

### Gene co-expression network construction, visualization, and analysis

Gene co-expression was measured using the correlation between gene expression profiles over the 24 h time series. Two daily rhythmic genes were assumed to be co-expressed when the Pearson correlation coefficient between their expression profiles was greater than 0.95. A gene co-expression network was constructed for each species where the nodes represent daily rhythmic genes and an edge is drawn between nodes when the corresponding genes are co-expressed according to the previous criterion. Cytoscape, a software tool for the representation and analysis of complex networks (Shannon et al., 2003), was used to visualize the gene co-expression networks applying the Prefuse Force-Directed Layout. The analysis of the networks was performed using the R package igraph (Csárdi and Nepusz, 2006). The scale-free property was tested using linear regression over the logarithmic transform of the degree distribution. The small-world property was tested by generating 10^4^ random scale-free networks with the same number of nodes and edges as the corresponding network, using the *barabasi.game* function from the igraph package.

### Module conservation analysis

Two daily rhythmic genes from two different species determined as potential orthologues using MBBH were defined as exhibiting a conserved daily pattern when both belonged to the same cluster (they presented their peak and trough in the same time interval) or when the Pearson correlation coefficient between their expression profiles were higher than 0.98. The conservation among the co-expression patterns of two different sets of genes from two different species were computed according to the *summary composite conservation statistic* “Zsummary” as defined in Langfelder et al. (2011). A Zsummary value lower than 2 indicates no conservation, a Zsummary value 2–10 implies moderate conservation, while a Zsummary greater than 10 constitutes evidence of a great level of conservation. The R package WGCNA (Langfelder and Horvath, 2012) and the function implemented therein were used.

### Plant, algal material, and growth conditions

Three independent biological replicates for plants and algae were grown in a model SG-1400 phytotron (Radiber SA, Spain) under LD conditions with temperatures ranging from 22°C (day) to 18°C (night) and 75 μEm^−2^s^−1^ light intensity. *Arabidopsis thaliana* Col-0 wild type seeds were incubated 4 days at 4°C in the dark before sowing in MS plates. 12-day-old seedlings were collected every 4 h during a 24 h period. Time points were denoted as Zeitgeber Time *N* (ZTN) indicating the time point *N* hours after the lights are switched on in the phytotron (ZT0), mimicking dawn. *Chlamydomonas reinhardtii* wild type CW15 was grown in flasks with minimal Sueoka medium for 12 days. Similarly, *Ostreococcus tauri* wild type RCC 745 was grown in flasks with Keller medium (Keller et al., 1987) for 12 days. Algae were harvested every 4 h during a 24 h period.

### RNA isolation and QPCR

RNA was isolated from *Arabidopsis* seedlings (0.1 g leaf tissue), *Chlamydomonas* and *Ostreococcus* (pellet of 25 ml culture at exponential phase) using a modified TRIZOL (Invitrogen) protocol as described by the manufacturer. Briefly, the sample was mixed with 1 ml of TRIZOL and 0.2 ml of chloroform and the mixture was centrifuged at 16,000 g for 10 min at 4°C. The supernatant was treated with 1 volume of 100% (v/v) 2-propanol, incubated 15 min at room temperature and centrifuged at 16,000 g for 10 min at 4°C. The pellet was dissolved in 0.75 ml 3 M LiCl, incubated for *t* > 10 min at room temperature and centrifuged at 16,000 g for 10 min at 4°C. This pellet was washed with 80% (v/v) ethanol and centrifuged at 16,000 g for 10 min at 4°C. The final RNA sample was suspended in 21 μl of DEPC treated water and 1 μl quantified employing a ND-1000 Spectrophotometer (Nanodrop).

One micro gram of TRIZOL-isolated RNA was used to synthesize cDNA employing the Quantitec® Reverse kit (Qiagen) following the instructions recommended by the manufacturer. cDNA samples were diluted to a final concentration of 10 ng/μL and stored at −20°C until QPCR was performed. Primers to amplify the 3′ translated region of *AtHY5, CrHY5, OtHY5*, including *AtUBQ10, CrTUB*, and *OtEF1*α as housekeeping genes (Table 1) were designed using the Oligo analyzer program from Integrated DNA Technologies (http://eu.idtdna.com/analyzer/Applications/OligoAnalyzer/). QPCR was performed in a Multicolor Real-Time PCR Detection System iQTM5 (Bio-Rad) in a 10 μL reaction: primer concentration 0.2 μM, 10 ng cDNA and 5 μL SensiFAST TM SYBR & Fluorescein Kit (Bioline). The QPCR program consisted in (i) 1 cycle (95°C, 2 min); (ii) 40 cycle (95°C, 5 s; 60°C, 10 s and 72°C, 6 s) (iii) 1 cycle (72°C, 6 s). Fluorescence was measured at the end of each extension step and the melting curve was performed between 55 and 95°C. Three biological replicates with three technical replicates from each species were used for every time point. The QPCR results were estimated using the ddCt R Bioconductor package (Zhang et al., 2015).

**Table 1:** Primers employed in QPCR experiments.

	**Forward sequence**	**Reverse sequence**	**Amplified fragment size (bp)**
***Arabidopsis*** **GENES**			
*UBQ10*	5′-GAAGTTCAATGTTTCGTTTCATGT-3′	5′-GGATTATACAAGGCCCCAAAA-3′	145
*HY5*	5′-GCTGAAGAGGTTGTTGAGGA-3′	5′-TCTCCAAGTCTTTCACTCTGTTT-3′	101
***Chlamydomonas*** **GENES**			
*CrTUB*	5′-GTTGCATCGTTAGCGTGGACG-3′	5′-GCAGCAGCCAATGTTCAGACT-3′	170
*CrHY5*	5′-GGCATGAACCCTAGCTTCC-3′	5′-CATCATGTCCTCGCTGATGT-3′	144
***Ostreococcus*** **GENES**			
*OtEF1α*	5′-GACGCGACGGTGGATCAA-3′	5′-CGACTGCCATCGTTTTACC-3′	202
*OtHY5*	5′-GAGGGAAGATTGGGAAGAAGAA-3′	5′-CCTTCTCCAACGCCTGAAT-3′	81

## Results and discussions

### Most *Ostreococcus* proteins present potential orthologues in *Arabidopsis* and *Chlamydomonas*, but this is not the case with the other two species

The identification of potential orthologues between two species constitutes one of the most important bottlenecks in comparative genomics and transcriptomics (Dessimoz et al., 2012). The Bidirectional Best Hit (BBH) algorithm is the most commonly used method for the automated identification of putative orthologues. In spite of its simplicity, BBH is highly accurate when dealing with bacterial and archaeal genomes (Wolf and Koonin, 2012). Nevertheless, BBH does not perform optimally, missing as much as 60% of orthologues, in gene-duplication-enriched genomes such as those of plants and animals (Dalquen and Dessimoz, 2013). In this work, in order to identify orthology among distantly related species, we have developed a variant of the BBH algorithm termed Multiple Bidirectional Best Hit (MBBH). MBBH assigns to each gene $g_{i}^{\text{1}}$ from the first species *k* ≤ *N* potential orthologues from the second species, $g_{1}^{\text{2}}$*,…,*$g_{k}^{\text{2}}$, based on sequence similarity of the proteins they encode if, and only if, the query gene $g_{i}^{\text{1}}$ is among the *N* most similar genes from the first species (Section Materials and Methods, Figure 1).

Using MBBH tool, more than 97% of *Ostreococcus* proteins could be ascribed to a potential orthologue either in *Arabidopsis* or *Chlamydomonas* (Supplementary Table 1). This is in agreement with the reduced and compact *Ostreococcus* genome (Palenik et al., 2007) and suggests, as has been proposed (Raven et al., 2013), that this marine picoeukaryote contains the minimal genome for a functional photosynthetic eukaryote and that it is almost entirely regulated by the circadian clock (see below). Confirming this idea, a GO term enrichment analysis over the set of genes without potential *Arabidopsis* or *Chlamydomonas* orthologues did not produce any significant result, indicating that these genes are not involved in any specific biological process. In fact, the highest number of genes without potential orthologues was located on chromosomes 2 and 19. Some of these genes codified for a number of unnamed products (ostta02g03690, ostta02g05500, ostta02g00800) and a group of Tc1-like/mariner transposases (ostta02g01245, ostta02g01247, ostta02g02355). This supports previous analysis on the heterogeneity of *Ostreococcus* genome that identified chromosomes 2 and 19 as different from the other chromosomes in terms of GC content, codon usage and number of transposable elements (Derelle et al., 2006).

**Figure 2:**
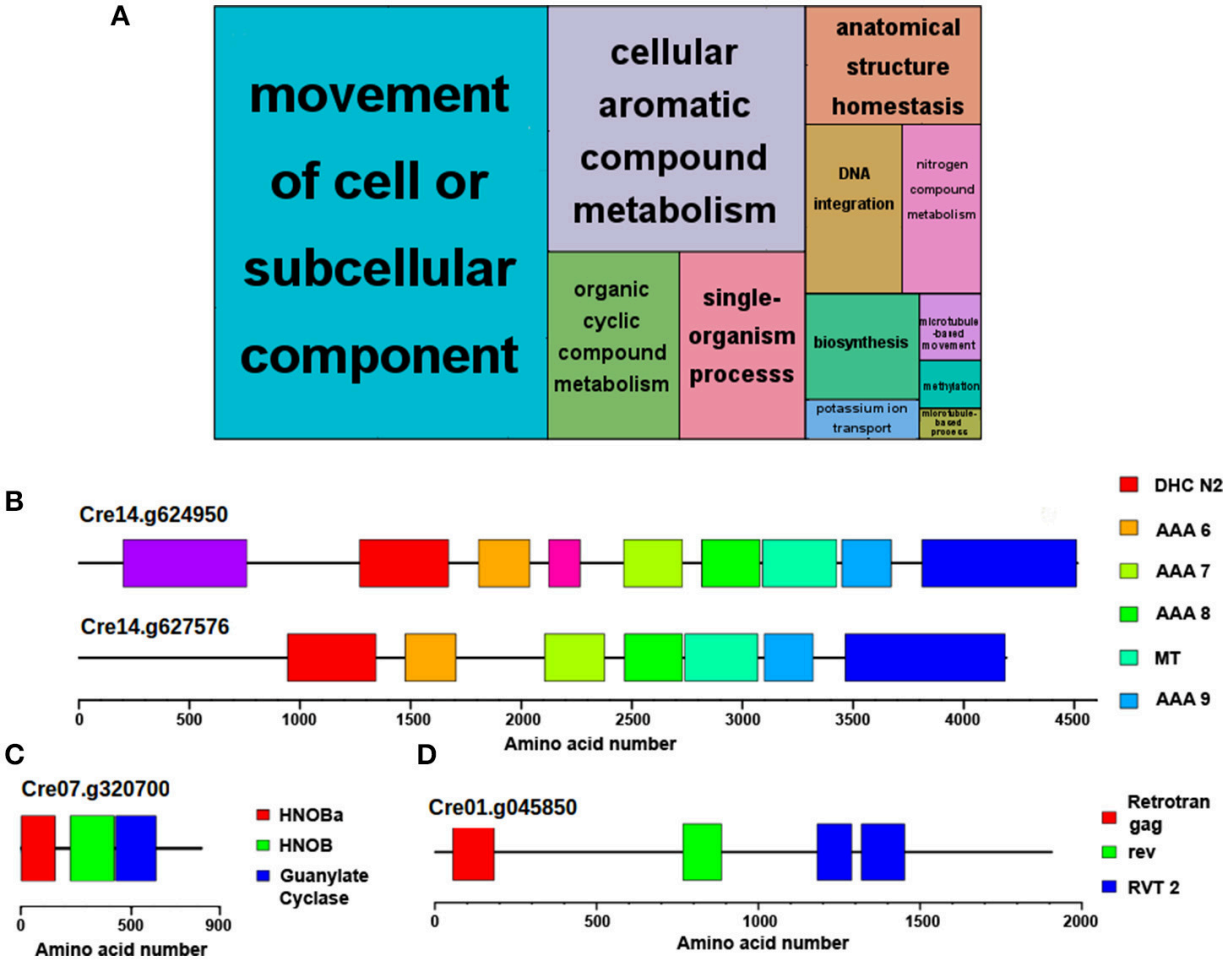
Functional annotation of genes exclusively identified in *Chlamydomonas*. No potential *Ostreococcus* or *Arabidopsis* orthologues were identified for 15% of *Chlamydomonas* proteins. **(A)** Non-redundant GO term enrichment analysis over the set of *Chlamydomonas* genes with no potential orthologue in the other species, suggesting that they are mainly involved in “*movement of cell or subcellular component*,” “*organic cyclic compound metabolism*,” and “*DNA integration*”. Each rectangle area in the treemap represents the −log10 (*p*-value) for the corresponding GO-term. **(B)** Domain structure of the proteins encoded by *Flagellar inner arm dynein 1* (Cre14.g624950) and *axonemal dynein heavy chain 6* (Cre14.g627576), two *Chlamydomonas*-specific flagellar proteins absent in *Ostreococcus* and *Arabidopsis* involved in “*movement of cell*” or “*subcellular component*.” **(C)** Domain structure of class III guanylyl and adenylyl cyclase encoded by *CYG11* (Cre07.g320700), a *Chlamydomonas*-specific protein involved in “*organic cyclic compound metabolism*.” This protein shows a higher homology to animal cyclases than to plant ones. **(D)** Domain structure of the protein encoded by *gag-pol-related retrotransposon* (Cre01.g045850), an example of *Chlamydomonas* specific proteins associated to the “*DNA integration*” GO term. Color boxes represent domains identified in pfam database including their identification codes.

Following the same comparative analysis, approximately 85% of *Chlamydomonas* proteins present potential orthologues in *Ostreococcus* and *Arabidopsis* (Supplementary Table 2). Therefore, a set of approximately 2,600 *Chlamydomonas* genes that codify for specific proteins could not be traced to any orthologue in the other two species. An ontology enrichment analysis performed over this set of genes using the R Bioconductor package topGO and summarized with REVIGO revealed that the top three most significant and non-redundant GO terms were “*Movement of cell or subcellular component*,” “*Organic cyclic compound metabolism*,” and “*DNA integration*” (Figure 2A). The same set of genes were associated with the GO terms “*Movement of cell or subcellular component*,” “*Anatomical structure homeostasis”* and “*single-organism process.”* Therefore, the last two GO terms were considered redundant and only “*Movement of cell or subcellular component*” is discussed. Similarly, the GO terms “*Organic cyclic compound metabolism”* and “*cellular aromatic compound metabolism”* included the same list of associated genes than “*Organic cyclic compound metabolism*,” so only the latter is discussed. Genes codifying flagellar proteins such as *flagellar inner arm dynein 1* (Cre14.g624950) and *axonemal dynein heavy chain 6* (Cre14g.627576) (Figure 2B) are representative of the group “Movement of cell or subcellular component.” *Chlamydomonas* cells exhibit two polar flagella whereas no flagellar-like structures are present in *Ostreococcus* or *Arabidopsis* cells, explaining the lack of orthologues in the non-flagellated organisms. Besides, the *Chlamydomonas* specific genes annotated with the GO term “*Organic cyclic compound metabolism”* include the class III guanylyl and adenylyl cyclase family including genes such as *adelynate/guanylate cyclase CYG11* (Cre07.g320700) (Figure 2C). These enzymes that catalyze the synthesis of cGMP and cAMP in animals are absent in plants, hence the lack of potential orthologues in *Ostreococcus* and *Arabidopsis*. In fact, this concurs with the idea that some *Chlamydomonas* genes are closer to animal than to plant ones (Merchant et al., 2007). On the other hand, the unique *gag-pol-related retrotransposon* (Cre01g.045850) (Figure 2D) is an example of the *Chlamydomonas* specific genes associated with “*DNA integration”* GO term, suggesting that this set of viral pathogens infecting *Chlamydomonas* is specific for the alga.

**Figure 3:**
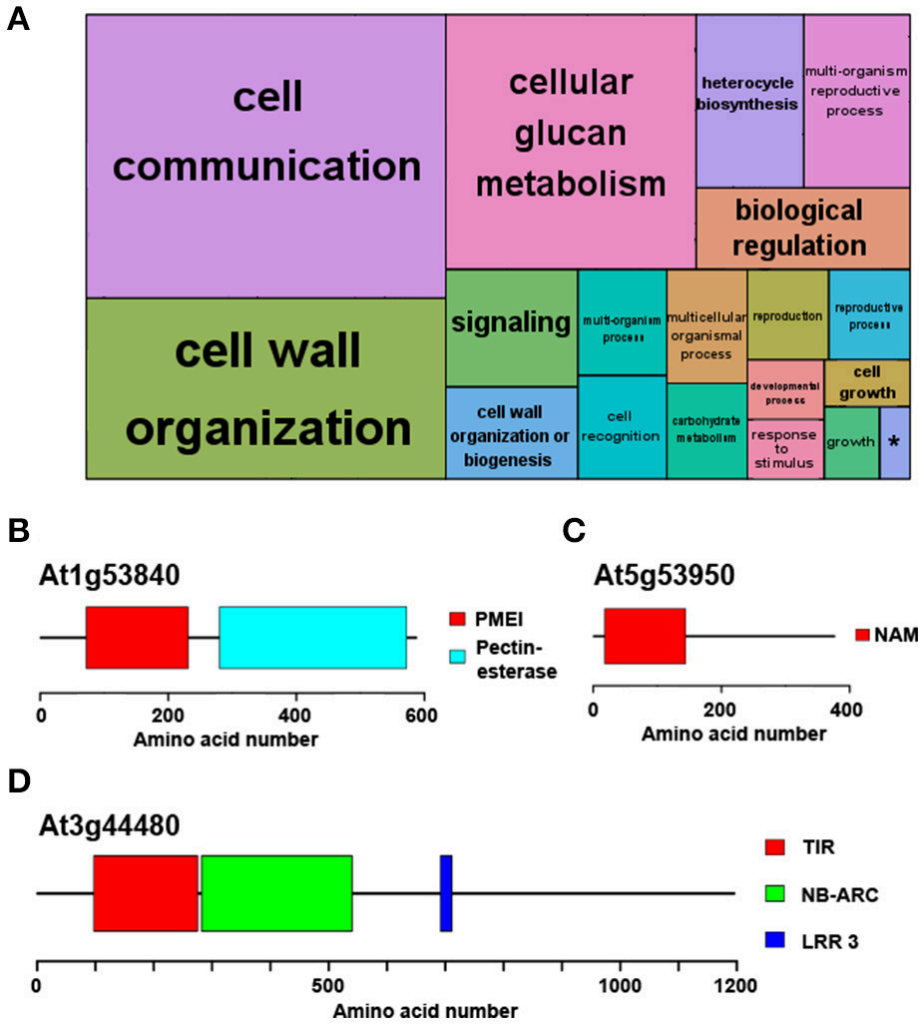
Functional annotation of genes exclusively identified in *Arabidopsis*. No potential *Chlamydomonas* or *Ostreococcus* orthologues were identified for a quarter of *Arabidopsis* proteins. **(A)** Non-redundant GO term enrichment analysis over the set of *Arabidopsis*-specific genes suggests that they are mainly involved in “*cell communication/signaling*,” “*cell wall organization*,” “*cellular glucan metabolism*” and “*multiorganism reproductive process*.” (^*^) “s*ingle-organism developmental process*.” Each rectangle area in the treemap represents the −log10 (*p*-value) for the corresponding GO-term. **(B)** Domain structure of the protein encoded by *Pectin Methylesterase 1*, (*PME1*, At1g53840), an example of an *Arabidopsis* specific protein involved in “*cell wall organization and glucan metabolism*.” **(C)** An instance of an *Arabidopsis* specific protein annotated with “*multi-organism reproductive process*” is the protein encoded by *NAC domain containing protein 98* (*ANAC098*, At5g53950) involved in floral development. **(D)** Domain structure of the protein encoded by *Recognition of Peronospora Parasitica 1* (*RPP1*, At3g44480), one of the specific *Arabidopsis* proteins involved in “*cell communication/signaling*.” Color boxes represent domains identified in pfam database including their identification codes.

The analysis also identified potential *Ostreococcus* and *Chlamydomonas* orthologues for approximately 75% of *Arabidopsis* proteins (Supplementary Table 3), suggesting that one fourth of the *Arabidopsis* proteins are not present in microalgae and have potentially been acquired during the course of higher plant evolution. In accordance with this idea, a GO term enrichment analysis showed that these *Arabidopsis*-specific proteins are involved in the following top three most significant non-redundant terms: “*Cell communication*,” “*Cell wall organization*,” and “*Multiorganism reproductive process*” (Figure 3A). Similar to the previous result, the GO term “Signaling” was found to share the same gene list with “Cell communication”; “Cellular glucan metabolism” presented the same associated genes as “Cell wall organization” and the set of genes assigned to “Biological regulation” and “Multiorganism reproductive process” were identical. Therefore, the former GO terms were considered redundant and only the latter GO terms are discussed. These GO terms correspond to complex multicellular plants features that are absent in microalgae. This way, key proteins involved in cell wall biogenesis and cellular glucan metabolism in *Arabidopsis* such as *Pectin Methylesterase 1* (*PME1*, At1g53840) lack *Ostreococcus* and *Chlamydomonas* potential orthologues (Figure 3B). Indeed, the evolutionary history of *PME* genes has been previously studied, establishing their appearance in multicellular Charophyte algae and supporting their absence in more primitive algae (Wang et al., 2013). A case of proteins annotated as involved in “*Multi-cellular processes*” without potential orthologues in *Ostreococcus* and *Chlamydomonas* is *Arabidopsis NAC domain containing protein 98* (*ANAC098*, At5g53950) (Figure 3C). The family of NAC TFs is one of the largest in plants and is involved in multiple key developmental processes such as floral development. Again, this TF family first originated in Charophytes (Zhu et al., 2012) and is absent in *Chlamydomonas* and *Ostreococcus*. Finally, the *Recognition of Peronospora Parasitica 1* (*RPP1*, At3g44480) is an example of a specific *Arabidopsis* protein involved in “*Cell communication/signaling*” and is part of the set of specific *Arabidopsis* genes related to pathogen resistance and programmed cell death (Schreiber et al., 2016; Figure 3D).

### Large TF families in *Arabidopsis* have single orthologues in microalgae suggesting gene amplification and functional diversification processes

The approach to define potential orthologues by MBBH can detect several candidates for any given gene, identifying multiple orthologous genes that could have appeared from processes of gene duplication. In *Arabidopsis*, on average, 5.19 and 7.83 genes could be ascribed to a homolog in *Chlamydomonas* and *Ostreococcus*, indicating that *Arabidopsis* gene families are, on average, five to eight times larger than in those organisms. This concurs with the idea that whole genome duplication and gene duplication events were crucial in the evolution of plant gene families (Romero-Campero et al., 2013; Rensing, 2014). Interestingly, this process is particularly frequent in TF families and MBBH tool has efficiently detected functional domains in the protein sequences of the three species under study and identified the TF family they belong to. In general, their sizes coincided with the data available in the Plant Transcription Factor Database, PlantTFDB (Jin et al., 2017), confirming the accuracy of this approach. On average, less than 4 and 7 protein members formed each *Ostreococcus* and *Chlamydomonas* TF family respectively, whereas a media of 30 members constituted *Arabidopsis* TF families, further supporting the idea of multiple duplication events.

**Figure 4:**
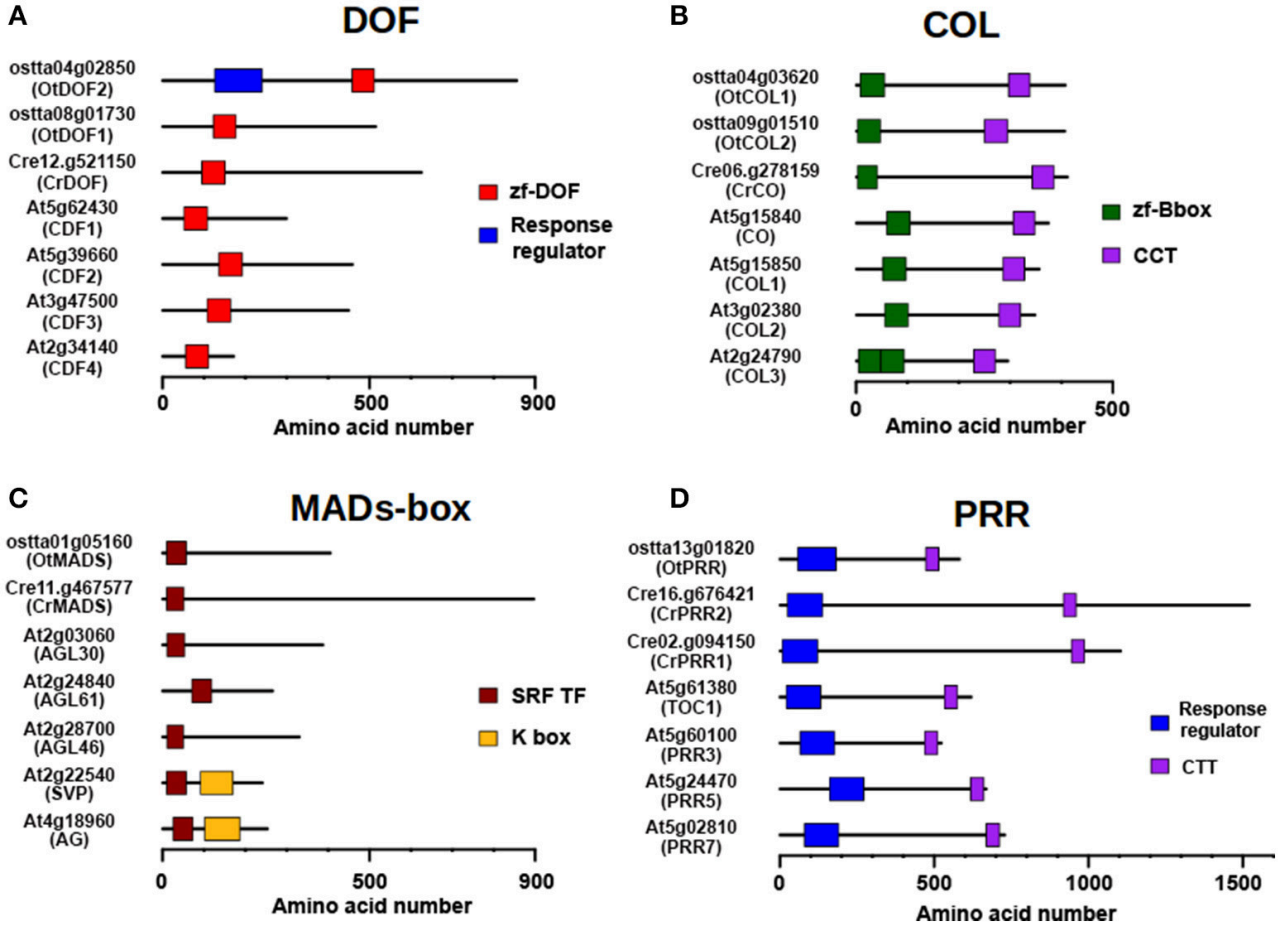
Amplification of TF families through evolution. *Arabidopsis* TF families are respectively on average five and eight times larger than *Chlamydomonas* or *Ostreococcus* ones, indicating an evolutionary history with multiple gene duplication events. **(A)** Domain structure of DOF proteins. *Ostreococcus* has two members; one of them exhibiting a Response Regulator domain in the N-terminal (OtDOF2), while it is constituted by a single member in *Chlamydomonas*. Of the 47 members of the family in *Arabidopsis*, the closest homologs to algal DOFs are the CDFs, involved in photoperiodic flowering. **(B)** Domain structure of the COL TF family. It has two members in *Ostreococcus*, a single member in *Chlamydomonas* and 22 in *Arabidopsis*. Some variation in the number of B-boxes in the N-terminal domain is observed. CO and closest homologs are shown. **(C)** Domain structure of the MADS-box TF family. This family has gone through an intense process of amplification from the single copy gene present in *Ostreococcus* and *Chlamydomonas* to the multi-gene family, constituted by 146 members, in *Arabidopsis*. The acquisition of novel protein domains in *Arabidopsis* would have given rise to different subfamilies. **(D)** Domain structure of PRR protein family. Although relatively small in *Arabidopsis*, with around 10 members, it has also been submitted to a process of gene amplification starting from the single copy gene in *Ostreococcus* and two genes in *Chlamydomonas*. Color boxes represent domains identified in pfam database including their identification codes.

Two clear examples of amplification in TFs are the DOF and COL protein families (Figures 4A,B) that present one single member in *Chlamydomonas* and two members in *Ostreococcus*, respectively, whereas in *Arabidopsis* 47 DOF and 22 COL proteins are present. A subset of plant *DOF* genes are regulated by the clock (*CYCLYNG DOF FACTORS, CDFs*) and, in time, control the daily expression of *CONSTANS* (*CO*) in *Arabidopsis* and potato during photoperiodic flowering (Imaizumi et al., 2005; Fornara et al., 2009; Kloosterman et al., 2013). This constitutes a conserved DOF-CO module in Spermatophytes that is also conserved in *Chlamydomonas* (Lucas-Reina et al., 2015). In *Ostreococcus* it might be different. While the *Chlamydomonas* and *Arabidopsis* orthologues presented a single DOF domain, one of the two *Ostreococcus* DOF proteins (OtDOF2, ostta04g02850) presented an additional N-terminal Response Regulator domain with a potential phosphoaceptor aspartic acid-aspartic acid-lysine (DDK) motif (Figure 4A). This suggests that OtDOF2 could be part of a phosphorelay system (Djouani-Tahri et al., 2011). The *COL* gene family (Figure 4B) seemed to have appeared in microalgae, with a single representative in *Chlamydomonas* (*CrCO*, Cre06.g278159) (Valverde, 2011) and two putative orthologues in *Ostreococcus* (*OtCOL1*, ostta04g03620 and *OtCOL2*, ostta09g01510). In *Chlamydomonas CrCO* is involved in the control of the cell cycle, starch synthesis and oil content (Serrano et al., 2009; Deng et al., 2015) and some of these functions have been conserved in some *Arabidopsis* orthologues (Romero-Campero et al., 2013; Ortiz-Marchena et al., 2014).

The MADS-box and PRR families (Figures 4C,D) constitute other interesting cases. MADS-box TF family is an example of frequent amplification over the course of plant evolution, since only a single copy is present in *Chlamydomonas* and *Ostreococcus*, whereas in *Arabidopsis* this family contains 146 members. MADS-boxes have been classified into different subfamilies depending on the recruitment of new protein domains besides the conserved MADS DNA-binding domain, such as the K-boxes (Figure 4C), and that may explain the ample functional diversity of these TF family in higher plants. On the other hand, modern plant PRRs (Figure 4D) contain an N-terminal Pseudo Response Regulator domain and a C-terminal CCT domain, but algal proteins are different. Spermatophytic PRRs are similar in size to COLs and, like them, seem to have experienced a similar amplification process from a single gene copy in *Ostreococcus* (*OtPRR*, ostta13g01820) and two genes in *Chlamydomonas* (*CrPPR1*, Cre02g.094150; *CrPRR2*, Cre16.g676421) to a multi-gene family in *Arabidopsis*. Nevertheless, while in *Ostreococcus* and *Chlamydomonas* proteins, a potential true phosphoaceptor DDK motif is present, suggesting that the algal proteins still retain part of the ancestral phosphorelay signaling mechanism, this motif is missing in the higher plant domain, constituting a “Pseudo” Response Regulator (Mizuno and Nakamichi, 2005; Satbhai et al., 2011).

**Figure 5:**
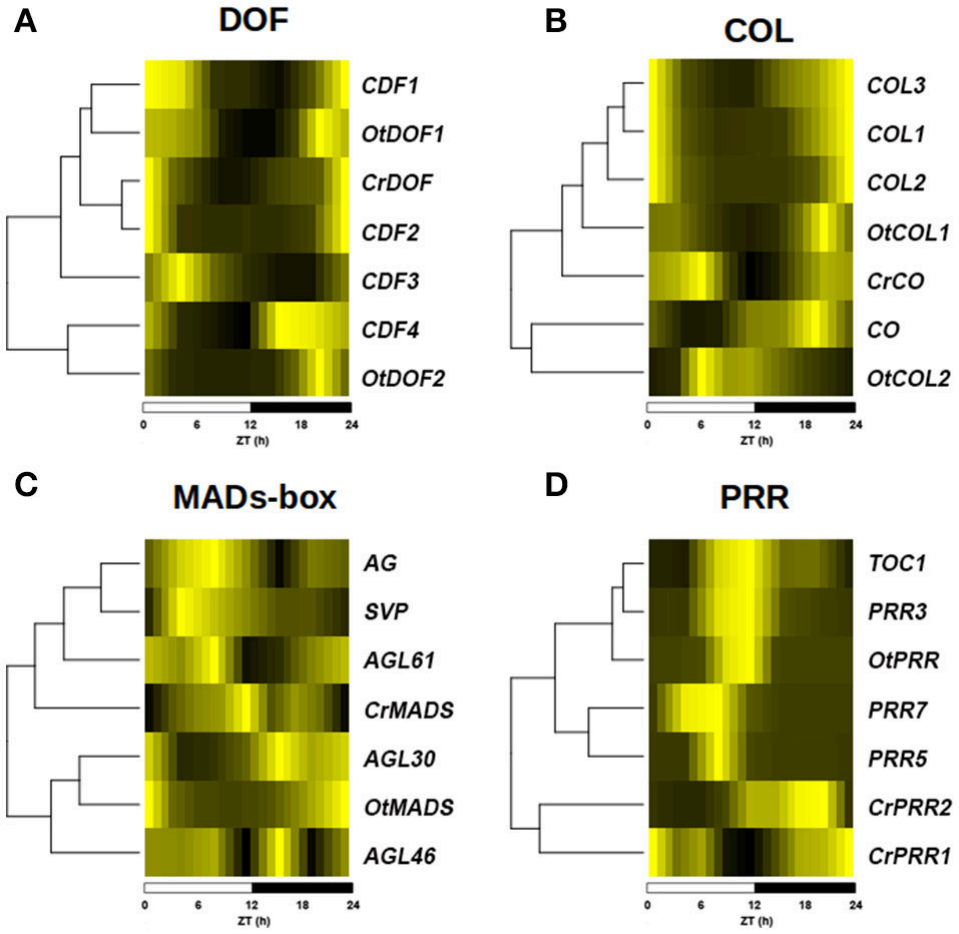
Diversification of expression profiles after gene amplification. Heat plots showing the diversification in the expression profiles of the different members of TF gene families from figure 4 in a ND photoperiod. A color scale from deep yellow (maximum expression) to black (minimal expression) is used. Hierarchical clustering is used to sort genes according to the similarity between their expression profiles. **(A)** Expression profile of *DOF* gene family. **(B)** Expression profile of *COL* gene family. **(C)** Expression profile of *MADS*-box gene family. **(D)** Expression profile of *PRR* gene family. Notice that while some *Arabidopsis* genes have retained similar expression patterns as *Ostreococcus* and *Chlamydomonas* genes, others have acquired completely new expression profiles. Below each graphic, a time scale in Zeitgeber Time (ZT) in hours (h) is shown.

To study to what extent the conservation in protein sequence and domain structure is accompanied by conservation in the expression profiles, transcriptomic data consisting of 24 h time series for the three species under study was analyzed. Not surprisingly, differences in the expression profiles of *Arabidopsis* genes were observed, some genes retaining the same one as in *Ostreococcus* and *Chlamydomonas*, while others acquired completely new expression profiles (Figure 5). This way, in the *DOF* family (Figure 5A) the expression profile of the *Arabidopsis* genes *CDF1* and *CDF2* have retained similar expression profiles as *CrDOF* and *OtDOF1* with a peak around ZT0 and a trough around ZT12. However, *CDF4* and *OtDOF2* peak around ZT21, while *CDF3* exhibits an expression profile with a peak at ZT3 and trough at ZT18, not observed in *Chlamydomonas* or *Ostreococcus* genes. A similar situation is observed for the COL (Figure 5B), MADS-box (Figure 5C) and PRR (Figure 5D) TF families. The *Arabidopsis COL* genes *COL1, COL2* and *COL3* exhibit very similar expression profiles to *OtCOL1* and *CrCO*, showing a trough at ZT12 (Figure 5B). On the other hand, *OtCO2* and *CO* present distinctly different expression profiles with their minimum expression levels at ZT0 and ZT6, respectively. The MADS-box family presents a great diversification in the expression profile of their members, with a substantial difference between the expression patterns of *Arabidopsis, Chlamydomonas* and *Ostreococcus* genes (Figure 5C). In the *PRR* family, *OtPRR* presents the same expression pattern as *Arabidopsis PRR1* (*TOC1*) and *PRR3*, peaking at ZT12, whereas *PRR5* and *PRR7* peak earlier, at around ZT9. However, *CrPRR1* and *CrPRR2* present completely different profiles, peaking at ZT21 and ZT0, respectively (Figure 5D). This could imply that, in general, gene amplification is accompanied by expression profile diversification and hence, functional diversification (Romero-Campero et al., 2013).

### Most genes in *Chlamydomonas* and *Ostreococcus* exhibit light-dependent daily rhythmic expression patterns, but only 40% of the *Arabidopsis* transcriptome shows a periodic expression

Recently, massive amounts of transcriptomic data for photosynthetic organisms under diverse environmental and physiological conditions have been produced. One of the most studied environmental signals is the alternation of light and dark cycles. Nevertheless, the analysis of the diurnal changes in the transcriptome has been independently performed in different species, making necessary the application of Molecular Systems Biology techniques to integrate and compare them. In this study we have analyzed microarray data collected over 24 h periods in ND conditions for *Ostreococcus* (Monnier et al., 2010) and *Arabidopsis* (Bläsing et al., 2005), as well as RNA-seq data for *Chlamydomonas* (Zones et al., 2015) in ND conditions and *Arabidopsis* in LD conditions (Rugnone et al., 2013). Details on transcriptomic data processing are described in the Materials and Methods section. In order to determine the effect of the alternation of light and dark periods over the transcriptome we identified genes exhibiting rhythmic or oscillating expression patterns with a period of approximately 24 h in the three species under study. Nonparametric methods implemented in the Bioconductor R package RAIN (Rhythmicity Analysis Incorporating Nonparametric methods) were used to detect significant gene expression patterns with arbitrary wave forms and a pre-specified period of 24 h (Thaben and Westermark, 2014), see Materials and Methods section for details.

The analysis revealed that more than 90% of the *Ostreococcus* genes targeted in the microarray exhibited daily rhythmic patterns (Supplementary Table 1). No GO term significantly enriched in the non-periodic genes was identified. This suggests that practically the entire *Ostreococcus* transcriptome is periodic and that most *Ostreococcus* biological processes are strongly affected by the alternation of light and dark cycles. In *Chlamydomonas* approximately 70% of the genes were found to follow significant daily rhythmic expression patterns (Supplementary Table 2). The GO term enrichment analysis over the non-periodic *Chlamydomonas* genes identified only a few significant biological processes. The two most significant processes were “*DNA integration”* and “*Defense response to virus*” including genes such as the reverse transcriptase Cre05.g235102, the DNA ligase Cre06.g277801 and the 2′-5′ oligoadenylate synthetase Cre15.g641050. These have been identified as biological processes induced by biotic stimuli, and are thus independent from the abiotic inputs from light/dark cycles. Finally, only 43.18% of the *Arabidopsis* genes showed significant circadian patterns according to our analysis (Supplementary Table 3), suggesting that during the evolution of higher plants many biological processes were uncoupled from the external influence of alternating light and dark cycles. These percentages of rhythmic genes are largely in agreement with previous results (Bläsing et al., 2005; Monnier et al., 2010; Zones et al., 2015). The pathway enrichment analysis performed over the *Arabidopsis* daily rhythmic genes revealed several key significant pathways influenced by rhythmic changes (Table 2). As expected, the most significant pathway represented in the enrichment was the one termed “*Circadian rhythms in plants*.” Also expected were the pathways “*Porphyrin and chlorophyll metabolism*” and “*Pentose phosphate pathway*”. These pathways are involved in photosynthesis and hence are expected to be highly regulated by light/dark cycles exhibiting rhythmic patterns. Key metabolic pathways such as “*Fatty acid degradation*” and “*Starch and sucrose metabolism*” were also detected as significantly enriched in daily rhythmic genes indicating that *Arabidopsis* metabolism is still highly affected by alternating periods of light and dark following rhythmic patterns. Somehow surprising was the identification of the “*Alpha-Linolenic acid metabolism*” and “*Plant hormone signal transduction*” among the enriched GO groups. This result suggests either that these pathways are ancient or that during the course of plant speciation new or exclusive plant pathways involved in hormone synthesis and sensing were recruited and acquired a daily rhythmic regulatory pattern. Curiously, although the course of evolution seems to have made the clock more complex in *Arabidopsis*, a lower percentage of the transcriptome seems to be regulated in a light-dependent periodic manner. It could be that a precise, fine tuning of the clock was recruited to make new processes independent from light/dark transitions in higher plants. More circadian experiments in different tissues or developmental stages may be needed to explain this apparent paradox.

**Table 2:** Significantly enriched pathways among the *Arabidopsis* circadian genes.

**Pathway ID**	**Description**	***q*-value**	**Ratio circadian/total**
ath04712	Circadian rhythm-plant	4.20 · 10^−6^	29/36
ath00030	Pentose phosphate pathway	5.25 · 10^−3^	35/58
ath00052	Galactose metabolism	1.54 · 10^−2^	32/55
ath00590	Arachidonic acid metabolism	1.54 · 10^−2^	13/17
ath00966	Glucosinolate biosynthesis	1.54 · 10^−2^	14/19
ath00860	Porphyrin and chlorophyll metabolism	1.76 · 10^−2^	28/48
ath00360	Phenylalanine metabolism	1.84 · 10^−2^	25/42
ath00071	Fatty acid degradation	2.66 · 10^−2^	24/41
ath00500	Starch and sucrose metabolism	2.66 · 10^−2^	66/139
ath00130	Ubiquinone and other terpenoid-quinone biosynthesis	2.66 · 10^−2^	21/35
ath00260	Glycine, serine and threonine metabolism	3.54 · 10^−2^	37/72
ath00592	alpha-Linolenic acid metabolism	3.54 · 10^−2^	21/36
ath01200	Carbon metabolism	3.62 · 10^−2^	114/262
ath01230	Biosynthesis of amino acids	3.62 · 10^−2^	111/255
ath04075	Plant hormone signal transduction	3.62 · 10^−2^	118/273
ath01210	2-Oxocarboxylic acid metabolism	4.66 · 10^−2^	37/74

**Figure 6:**
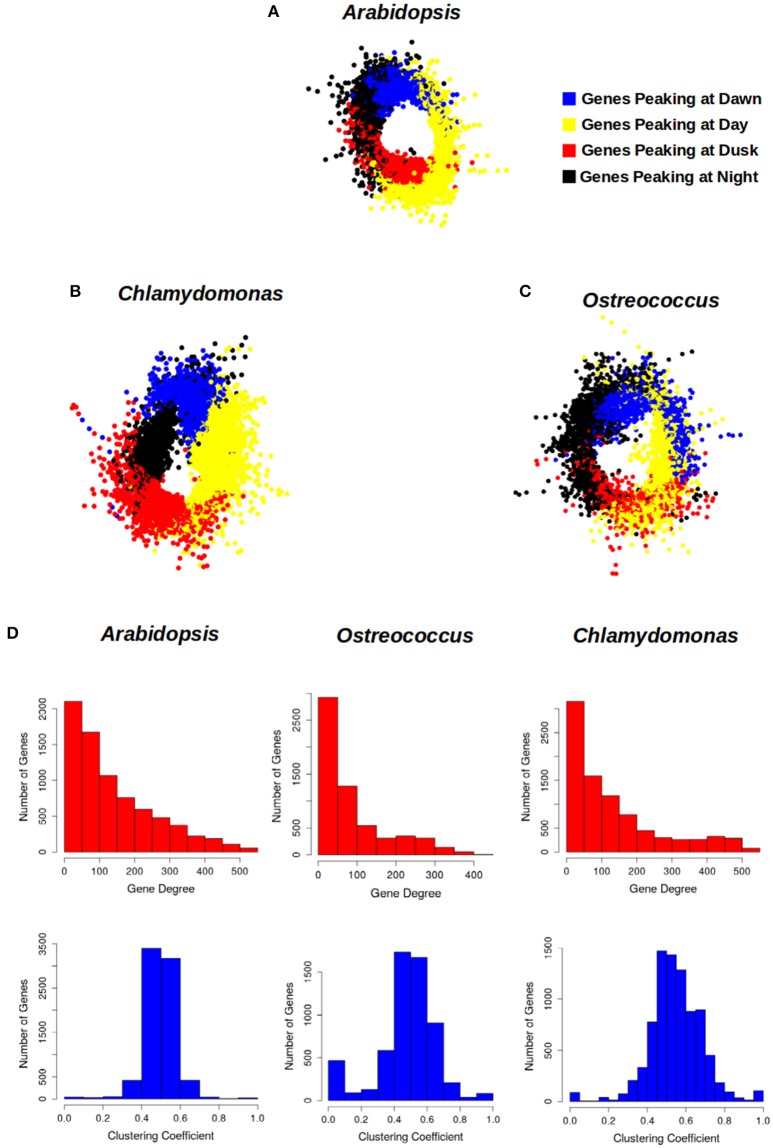
Circadian gene co-expression networks. Genes are represented by nodes and edges represent co-expression between them (Pearson correlation index between their 24 h-expression profiles greater than 0.95). The three networks acquired a ring-like structure mirroring the periodic expression pattern of daily rhythmic genes. **(A)** *Arabidopsis* daily rhythmic gene co-expression network comprised 7639 circadian genes with 537027 co-expression relationships. **(B)** *Chlamydomonas* daily rhythmic gene co-expression network consisted of 10338 circadian genes and 573980 co-expression interactions. **(C)** *Ostreococcus* daily rhythmic gene co-expression network comprised 5782 nodes and 222493 co-expression relationships. **(D)** The topological analysis of the three gene co-expression network shows that they are scale-free and small-world networks: (1) Their degree distributions (above, red) follow an exponential negative distribution. (2) Their average clustering coefficient (below, blue) is significantly greater than random networks with equal number of nodes and edges. Gene cluster with a peak at Dawn is represented in blue, that with a peak during the Day in yellow, the one showing a peak at Dusk in red and that with a peak at Night in black.

With the aim of representing the daily rhythmic genes and their complex co-expression relationships, a gene co-expression network for each photosynthetic species analyzed in this study was constructed (Figures 6A–C). Nodes represent daily rhythmic genes and edges co-expression relationships, so that two circadian genes are assumed to be co-expressed when the Pearson correlation index between their 24 h expression profiles is greater than 0.95 and an edge is drawn between them. The three gene co-expression networks were visualized using the Prefuse Force Directed layout implemented in the software tool Cytoscape (Shannon et al., 2003). Interestingly, the three different gene co-expression networks acquired the same ring-like structure, capturing the chronological relationship between the co-expression patterns of the periodic genes. The basic topological parameters in network analysis, namely node degree distribution and clustering coefficient, were computed for each network (Figure 6D). The degree distribution of the three networks follows an exponential negative distribution with *p*-values below 2.2 · 10^−16^, which implies that they are scale-free networks (Barabási, 2015), suggesting that the global daily rhythmic co-expression patterns in the three species are robust to random changes or mutations but fragile to direct changes or mutations in hub genes, those co-expressed with a high number of genes (Aoki et al., 2007). Additionally, the clustering coefficient of the three networks was significantly high when compared to the clustering coefficient of random scale-free networks with the same number of nodes and edges. Therefore, all three networks constitute small-world networks (Barabási, 2015) with expected short paths connecting any two nodes of the network. This is assumed to facilitate the quick propagation of information between genes with different periodic expression patterns.

### Daily rhythmic genes clusters constitute transcriptional programs that confer temporal separation to different biological processes with different levels of conservation between *Ostreococcus, Chlamydomonas*, and *Arabidopsis*

**Figure 7:**
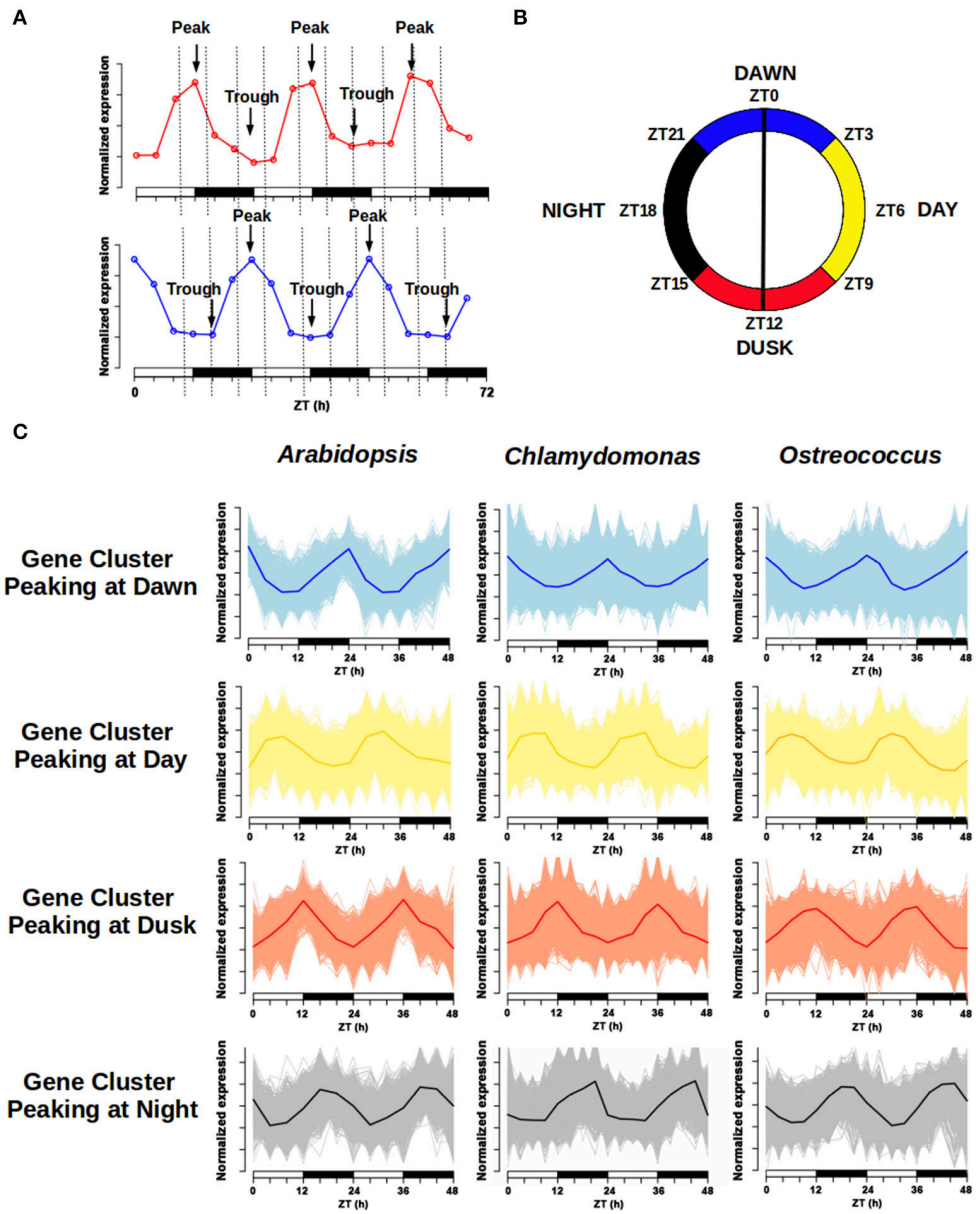
Daily rhythmic gene clustering. The wave form of daily periodic genes expression profile can be characterized by its peak (maximum) and trough (minimum). **(A)** Example of gene expression for two daily rhythmic genes in a 72 h course. Time is shown in hours (h) as Zeitgeber Time (ZT). The first one exhibits its peak around the light/dark transition and its trough around the dark/light, while the second one presents a symmetric profile. **(B)** The 24 h day was divided into four different time intervals: “Dawn” was defined as the time interval from ZT21 to ZT3 (blue); “Day” was considered from ZT3 to ZT9 (yellow); “Dusk” consisted of the time period from ZT9 to ZT15 (red) and “Night” was assumed to be from ZT15 to ZT21 (black). **(C)** Four different clusters were defined for visualization purposes following the same color code: genes peaking at Dawn, Day, Dusk, and Night. A 48 h course normalized expression profile of every gene in each cluster was represented, as well as the average gene expression profile for each cluster. Note that the wave form of the average expression profile for each gene cluster is similar in the three different species.

The daily rhythmic expression pattern of individual genes can be described by using its peak, the time point when the expression level is highest and its trough, the time point when the expression level is lowest (Figure 7A, Supplementary Tables 1–3). The transcriptomic data used in the co-expression networks was collected in ND conditions, but using different time sets. While *Ostreococcus* and *Chlamydomonas* data were collected every 3 h, *Arabidopsis* data were collected every 4 h. In order to compare the expression profiles from the different species, a 24 h day was divided into four temporal intervals that contained all points from the different time series. Therefore, Dawn (blue) was defined as the time interval from ZT21 to ZT3; Day (yellow) was considered from ZT3 to ZT9; Dusk (red) consisted of the time period from ZT9 to ZT15 and Night (black) was assumed to be from ZT15 to ZT21 (Figure 7B). Thus, daily rhythmic genes could be classified into 16 different clusters according to the time interval where their peak was located (Dawn, Day, Dusk, and Night) and the time interval containing their trough (again Dawn, Day, Dusk, and Night). Due to the amount of data engaged, the graphic representation of these 16 clusters can be confusing, so, in order to facilitate their visualization, we decided to merge all clusters peaking at the same interval into a single one. This way, for each generalized gene cluster we represented the expression profile of every gene and the average expression profile of the entire cluster (Figure 7C). Interestingly, no major differences were apparent between the average expression profiles from the three different species under analysis. Furthermore, when we colored the location of these clusters in the corresponding gene co-expression networks (Figures 6A–C), we observed that these clusters presented the same localization in the three different networks. This is a strong support for the veracity of our approach and shows, at a single view, that daily rhythmic genes associate in true temporal sets of genes performing synchronous actions and roughly reflecting the natural course of a 24 h clock. In this analysis the boundaries separating clusters were more sharply defined in *Arabidopsis* and *Chlamydomonas* than *Ostreococcus* that showed a higher degree of genes mixing between temporal clusters (Figures 6A–C). This supports the already established idea that the primitive *Ostreococcus* clock is not as efficient as those of the other more complex organisms.

The two largest gene clusters in *Ostreococcus* and *Chlamydomonas* contain genes peaking at Dusk with their troughs at Dawn (1620 and 3751 genes, respectively) and genes peaking at Dawn with their troughs at Dusk (1744 and 2869 genes, respectively; Table 3). The genes of these two clusters comprise 56.85 and 49.98% of the entire set of daily rhythmic genes in *Ostreococcus* and *Chlamydomonas*, respectively and concur with the common notion that the light/dark and dark/light transitions play central roles in daily rhythmic gene expression regulation in both algae. Surprisingly, in *Arabidopsis* these two gene clusters are not the largest ones, comprising only 22.51% of all periodic genes. This suggests a degree of uncoupling between regulation of circadian gene expression and light/dark and dark/light transitions in higher plants. Nevertheless, the largest gene cluster in *Arabidopsis* is made up of 1209 genes peaking at Dawn with their troughs in the Day, indicating that the dark/light transition still plays a key role in the regulation of daily rhythmic genes in *Arabidopsis*. The second largest cluster in *Arabidopsis* contains 1014 genes whose expression peaks take place at Night and troughs during the Day. This suggests certain independence in the regulation of daily rhythmic gene expression in *Arabidopsis* from the light/dark transitions since a large number of genes present their peaks or troughs in the periods of light and dark.

**Table 3:** GO term enrichment in the circadian gene clusters.

	***Arabidopsis thaliana***			***Ostreococcus tauri***			***Chlamydomonas reinhardtii***		
	**Size**	**Enriched GO terms**	**Genes**	**Size**	**Enriched GO terms**	**Genes**	**Size**	**Enriched GO terms**	**Genes**
Peak dawn	0			422	Protein catabolic process	*ostta11g00020*	1,426	Carboxylic acid metabolic process	*Cre16.g687350*
Trough dawn					Transcription from RNA polymerase II promoter	*ostta14g01300*		Cellular amino acid metabolic process	*Cre01.g029250*
Peak dawn	1,209	Carbohydrate catabolic process	*AT4G17090*	1,384	RNA metabolic process	*ostta10g00100*	1,801	Ribosome biogenesis	*Cre13.g573050*
Trough day		Monocarboxylic acid metabolic process	*AT4G25700*		Ribosome biogenesis	*ostta06g01560*		RNA metabolic process	*Cre01.g012350*
Peak dawn	811	Response to auxin	*AT1G29440*	1,744	Tetrapyrrole metabolic process	*ostta17g00130*	2,869	Tetrapyrrole metabolic process	*Cre06.g306300*
Trough dusk		Carbohydrate biosynthetic process	*AT1G70290*		Carbohydrate catabolic process	*ostta01g03040*		Carbohydrate catabolic process	*Cre01.g006950*
Peak dawn	132	Regulation of transcription	*AT5G62430*	739	Protein localization to membrane	*ostta03g05390*	1,212	Protein targeting to ER	*Cre16.g683950*
Trough night		Organic cyclic compound metabolic process	*AT1G20330*		Nucleotide metabolic process	*ostta15g00150*		Cyclic nucleotide metabolic process	*Cre02.g074150*
Peak day	980	DNA replication	*AT4G02060*	997	DNA metabolic process	*ostta03g03780*	1,987	Protein catabolic process	*Cre12.g531100*
Trough dawn		Actin cytoskeleton organization	*AT1G13180*		Establishment of localization	*ostta02g01890*		Golgi vesicle transport	*Cre10.g447350*
Peak day	127			141	Protein localization to membrane	*ostta01g00410*	256	Peptide biosynthetic process	*Cre10.g421600*
Trough day					Response to stress	*ostta08g03450*		Vitamin metabolic process	*Cre02.g090150*
Peak day	222	Tricarboxylic acid metabolic process	*AT5G04950*	514	Tetrapyrrole biosynthetic process	*ostta02g02380*	962	Photosynthesis	*Cre10.g425900*
Trough dusk		Isoprenoid metabolic process	*AT4G15560*		Photosynthesis	*ostta14g00150*		Tetrapyrrole biosynthetic process	*Cre06.g306300*
Peak day	935	Photosynthesis	*AT4G10340*	1,060	Signal transduction	*ostta04g01810*	1,467	Photosynthesis	*Cre16.g673650*
Trough night		Ribosome biogenesis	*AT5G16750*		DNA replication	*ostta01g02580*		Carbohydrate derivative	*Cre12.g508700*
					Photosynthesis	*ostta14g00150*		Biosynthetic process	
Peak dusk	907	DNA metabolic process	*AT2G42120*	1,620	DNA metabolic process	*ostta02g00690*	3,751	DNA metabolic process	*Cre01.g017450*
Trough dawn		Carbohydrate catabolic process	*AT2G32290*		Carbohydrate metabolic process	*ostta05g04330*		Carbohydrate biosynthetic process	*Cre12.g508700*
Peak dusk	432	Vitamin metabolic process	*AT2G29630*	437	Microtubule-based process	*ostta10g00680*	1,573	Mitotic cell cycle	*Cre02.g086650*
Trough day		Peroxisome organization	*AT1G47750*					Microtubule-based process	*Cre10.g456350*
Peak dusk	196	Peptide biosynthetic process	*AT4G29430*	696	Vesicle-mediated transport	*ostta09g03760*	882	Vesicle-mediated transport	*Cre02.g087551*
Trough night					DNA replication	*ostta04g04640*		Chromatin organization	*Cre16.g668200*
Peak nightt	122	Hexose metabolic process	*AT1G24280*	540	RNA processing	*ostta11g03270*	1,488	Phosphorylation	*Cre10.g423200*
Trough dawn					Protein modification process	*ostta11g02830*		Nucleotide biosynthetic process	*Cre13.g606250*
Peak night	1,014	Dephosphorylation	*AT1G71860*	1,234	Ribosome biogenesis	*ostta09g00820*	1,479	Protein metabolic process	*Cre01.g035500*
Trough day		Autophagy	*AT3G60640*		Vitamin metabolic process	*ostta09g00820*		Regulation of cellular process	*Cre12.g489000*
Peak night	177	Hexose metabolic process	*AT5G35790*	689	Gene expression	*ostta18g01220*	951	Protein metabolic process	*Cre01.g001400*
Trough dusk		Response to stimulus	*AT1G10470*		Peptide biosynthetic process	*ostta02g01970*		Signal transduction	*Cre13.g571200*
Peak night Trough night	3			60	L-serine metabolic process	*ostta12g03060*	57	Cellular respiration	*Cre13.g568800*

We performed GO term enrichment analysis over the different gene clusters (Table 3) in order to determine the biological processes that were carried out by the different rhythmic gene clusters. Different clusters were enriched in distinct biological processes indicating a temporal separation among them. Some biological processes show enrichment in the same clusters for the three different species such as “*DNA metabolic process*” (gene clusters with peak at Dusk and trough at Dawn), “*photosynthesis*” (gene clusters with peak at Day and trough at Night) and “*carbohydrate catabolic process*” (gene clusters that have the peak during the Dawn and trough at Dusk) indicating a conservation in their daily rhythmic expression patterns (Table 2). On the contrary, an anticipation, delay or uncoupling from periodic expression of certain biological processes was apparent when the three species were compared.

**Figure 8:**
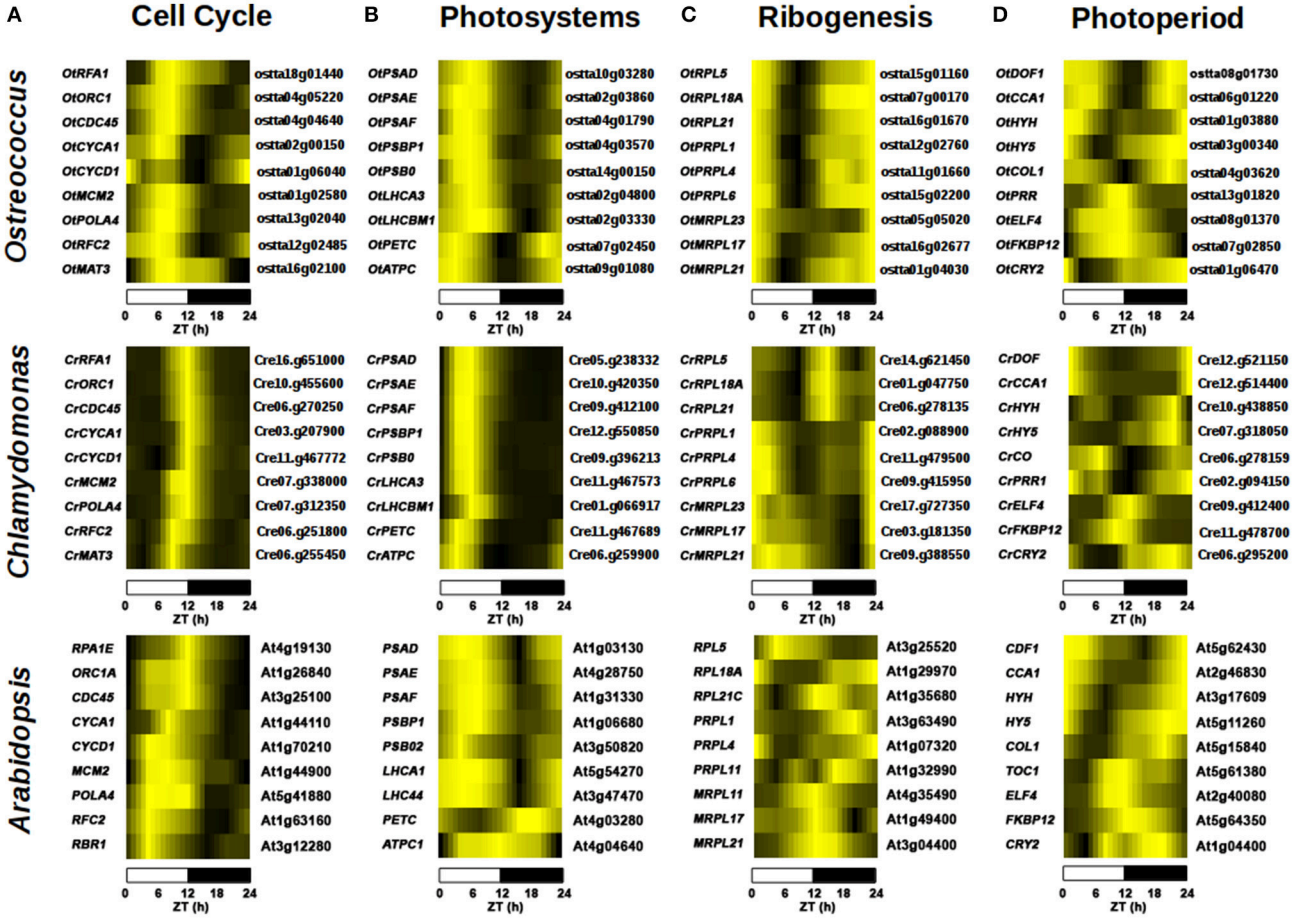
Comparison of gene expression patterns in key biological processes in *Chlamydomonas, Ostreococcus* and *Arabidopsis*. Heatmaps are used to represent gene expression with genes sorted according to their expression profile similarity using hierarchical clustering as in Figure 5. **(A)** Cell cycle genes such as *REPLICATION PROTEIN A* (*RPA*) and *ORIGIN RECOGNITION COMPLEX 1* (*ORC1*) exhibit a peak at Dusk and trough at Dawn in the three species. In *Ostreococcus* and *Arabidopsis* genes such as *DNA POLYMERASE A4* (*POLA4*) and *MINI CHROMOSOME MAINTENANCE 2* (*MCM2*) are expressed previously to those in *Chlamydomonas*, presenting broader peaks expanding from Day to Dusk with respect to the narrow peaks at Dusk observed in *Chlamydomonas*. **(B)** Photosystem components such as *PHOTOSYSTEM I SUBUNIT D* (*PSAD*) and *PHOTOSYSTEM II SUBUNIT O* (*PSBO*) follow similar expression patterns in the three species with peaks at Day and troughs at Night. Only small variations were detected in photosynthesis-involved genes such as the expression profiles of *ATP CHLOROPLAST SYNTHASE 1* (*ATPC1*) and *PHOTOSYNTHETIC ELECTRON TRANSFER C* (*PETC*). These genes present a peak at Dawn and trough at Dusk in both algae, while in *Arabidopsis, ATPC1* is delayed, peaking during the day and *PETC* anticipates dawn peaking at night. **(C)** Ribogenesis-involved genes present their peaks at Dawn and troughs at Day in the algae, such as *PLASTID RIBOSOMAL PROTEIN L1* (*PRPL1*) and *MITOCHONDRIAL RIBOSOMAL PROTEIN L21* (*MRPL21*). Cytosolic ribogenesis is uncoupled from organella ribogenesis in *Chlamydomonas* with genes such as *RIBOSOMAL PROTEIN L5, L21* (*RPL5, RPL21*), peaking at Dusk. This uncoupling is not evident in *Ostreococcus* and no apparent synchronization is observed between genes codifying for ribosome components in *Arabidopsis*. **(D)** Photoperiod/circadian central transcriptional regulators exhibit highly similar expression profiles in the three species. *DOF, CCA1*, and *COL* present the same expression pattern; peaking at Dawn with their troughs at Dusk. Some genes, such as *OtPRR* and *TOC1*, present very similar profiles (peaking at Dusk with their troughs at Dawn) in *Ostreococcus* and *Arabidopsis*, whereas in *Chlamydomonas CrPRR* follows a symmetric pattern peaking at Dawn and a trough at Dusk.

Cell cycle-related GO terms such as “*DNA metabolic process*” were enriched in the gene cluster with peak at Dusk and trough at Dawn in the three species, including genes such as *REPLICATION PROTEIN A* (Cre16.g65100, ostta18g01440 and At4g19130), *ORIGIN RECOGNITION COMPLEX 1* (Cre10.g455600, ostta04g05220, and At1g26840) and *CELL DIVISION CYCLE 45* (Cre06.g270250, ostta04g04640, and At3g25100) that showed very similar expression profiles in the three species (Figure 8A). Interestingly, the GO terms “*DNA replication*” and “*DNA metabolic process*” are also enriched in the gene cluster with peak at Day and trough at Dawn only in *Ostreococcus* and *Arabidopsis* indicating an apparent anticipation or broader gene expression peaks in these two species with respect to *Chlamydomonas*, where narrow peaks are observed at the light/dark transition (Figure 8A). For instance, *DNA POLYMERASE A4* (Cre07.g312350, ostta13g02040, and At5g41880), *MINI CHROMOSOME MAINTENANCE 2* (Cre07.g338000, ostta01g02580, and At1g44900), *CYCLIN A1* (Cre03.g207900, ostta02g00150, and At1g44110) present a narrow peak centered on ZT12 in *Chlamydomonas*, whereas in *Ostreococcus* and *Arabidopsis* they present a broader peak centered on ZT6. This could also indicate a better culture synchronization in *Chlamydomonas* than *Ostreococcus* and the lack of synchronicity between the different tissues in *Arabidopsis*. The GO term “*Photosynthesis*” appeared significantly enriched in the gene clusters with peak at the Day and trough at Night in the three different species (Table 3). Specifically, *PHOTOSYSTEM I SUBUNIT D* (Cre05.g238332, ostta10g03280, and At1g03130) and *PHOTOSYSTEM II SUBUNIT O* (Cre09.g396213, ostta14g00150, and At3g50820) exhibit very similar expression patterns with broader peaks in *Ostreococcus* and *Arabidopsis* than in *Chlamydomonas* (Figure 8B). Only small variations were detected, for instance, in the expression profiles of genes codifying for components of plastid ATP synthase and b6f complex. *ATP CHLOROPLAST SYNTHASE 1* (*ATPC*, Cre06.g259900, ostta09g01080, and At4g04640) and *PHOTOSYNTHETIC ELECTRON TRANSFER C* (*PETC*, Cre11g.467689, ostta07g02450, and At4g03280) present a peak at dawn and trough at dusk in *Chlamydomonas* and *Ostreococcus*, while in *Arabidopsis ATPC1* is delayed, peaking during the day and *PETC* anticipates dawn, peaking at night (Figure 8B).

Ribogenesis, the process of ribosome making, is another interesting example of conservation and evolution of the periodic pattern of a biological process. In fact, “*Ribosome biogenesis*” seems to be enriched mainly in the gene cluster with peak at Dawn and a trough during the Day in *Ostreococcus* and *Chlamydomonas*, whereas in *Arabidopsis* is only significantly detected in the gene cluster with peak at Day and trough at Night (Table 3). A closer inspection into ribogenesis in *Chlamydomonas* reveals an uncoupling between the genesis of cytosolic, plastid and mitochondrial ribosomes (Zones et al., 2015). Cytosolic ribosome components peak at Dusk (i.e., *CrRPL5*, Cre14.g621450), plastid ribosome components peak at Dawn (i.e., *CrPRPL1*, Cre02.g088900) and mitochondrial ribosome component present a broad peak at Dawn (i.e., *CrMRPL21*, Cre09.g388550) (Figure 8C). This uncoupling is less evident in *Ostreococcus* where genes codifying for components of cytosolic (i.e., *OtRPL5*, ostta15g01160), plastid (i.e., *OtPRPL1*, ostta12g02760) and mitochondrial (i.e., *OtMRPL21*, ostta01g04030) ribosomes exhibit a peak at Dawn and a trough at Day (Figure 8C). No apparent synchronization is observed between genes codifying for ribosome components in *Arabidopsis* (Figure 8C). Therefore, the analysis shows how the light/dark cycles in ancient algae synchronized all ribosome synthesis at the same time point. In *Chlamydomonas* ribogenesis was divided into three different stages depending on the nuclear, chloroplast or mitochondrial genome control, while *Arabidopsis* showed a ribosome biogenesis almost independent from daily rhythms.

The conservation and evolution of the central transcriptional regulators in circadian/photoperiod response in *Arabidopsis* was analyzed. A strong conservation was observed between the expression profiles of the key circadian/photoperiod regulators in *Ostreococcus* and *Arabidopsis* with some variations in *Chlamydomonas* (Figure 8D). For instance, the three different species showed similar expression profiles of two central photoperiodic genes; *DOF* (Cre12.g51440, ostta04g02850, At5g62430) and *COL* (Cre06.g278159, ostta04g03620, At5g15840) peaking at Dawn, with their trough at Dusk (Figure 8D). Remarkably, two of the central genes in the circadian clock; *CCA1* (At2g46830, ostta06g01220) and *TOC1* (At5g61380, ostta13g01820) exhibit the same symmetric expression profiles in *Ostreococcus* and *Arabidopsis*, while in *Chlamydomonas* the putative orthologues *CrCCA1* (Cre12.g514400) and *CrTOC1* (Cre02.g094150) detected in our analysis present the same expression profile, peaking at Dawn with their troughs at Dusk (Figure 8D). This suggests an independent and divergent evolution in *Chlamydomonas* of the core regulatory circuit of circadian rhythms as previously suggested (Mittag et al., 2005). Nevertheless, the full validation of these results would require more extensive analysis and experimental work.

Other relevant genes involved in photomorphogenesis such as the bZIP TF *ELONGATED HYPOCOTYL 5* (*HY5*) and *HY5-HOMOLOG* (*HYH*) exhibit the same expression pattern in *Chlamydomonas* (Cre07.g318050, Cre10.g438850), *Ostreococcus* (ostta03g00340, ostta01g03880), and *Arabidopsis* (At5g11260, At3g17609), peaking at Dawn with their troughs at Day (Figures 8D, **11C,D**).

**Figure 9:**
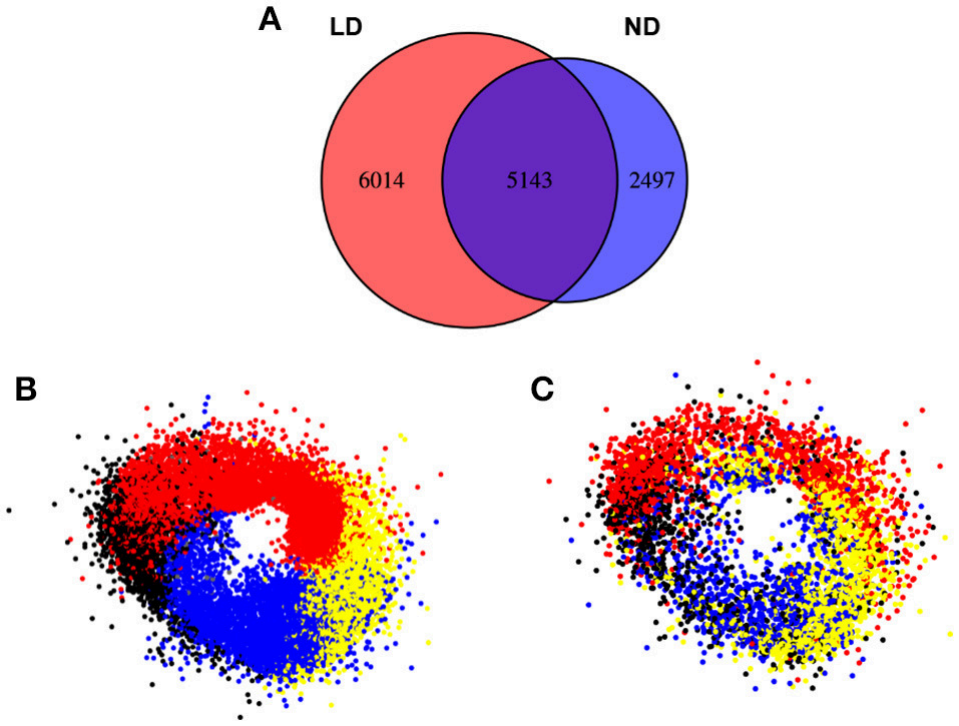
The clustering of the clock-regulated genes does not change substantially between ND and LD conditions in *Arabidopsis*. **(A)** Venn diagrams representing genes in LD (red) and ND (blue) clusters. In LD conditions a higher percentage of genes (41%) follow a circadian expression than in ND (33%). Most genes identified as circadian in ND conditions (7640) exhibit circadian patterns also in LD (5143, 76%). Nevertheless, new genes are identified as circadian in LD (6014, 55%) indicating a higher circadian dependence in LD. **(B)** In the gene co-expression network composed of circadian genes in LD (11157), the same ring-like structure as in ND conditions is observed. This is constituted by the sequential distribution of the clusters constituted by genes peaking at Dawn (red), Day (yellow), Dusk (blue), and Night (black). **(C)** In the representation of the clusters obtained in ND conditions intersecting with the gene co-expression network in LD conditions (deep purple, 5143 genes), an apparent conservation of the circadian pattern is observed.

It was also interesting to observe how the clustering approach worked when the photoperiod changed from ND to LD (Figure 9). This way, when the expression profile by RNAseq of a 24 h Col-0 plant grown in LD (red) was compared with the profile in ND (blue), more than 41% of the total genes expressed in LD presented a daily rhythmic profile (11157), while the percentage was reduced to 37% of genes in ND (7640). Out of the 7640 genes showing a periodic regulation in ND, 5143 (deep purple, 67%) could be also identified in LD, while approximately half of the 11157 genes in LD (6014, 54%) did not follow a daily rhythmic pattern in ND. This could mean that increasing the photoperiod also augments the daily rhythmic regulation of the transcriptome in higher plants, although never reaching the numbers shown in microalgae. When we organized the periodic-expressed genes in clusters, based on their peak and trough during the day as in Figure 8, a clear clock-wise distribution was observed (Figure 9B). This seems to indicate that the basic organization of the daily rhythms is independent of the photoperiod. However, when a temporal-clustered gene co-expression network was constructed with the intersection genes between daily rhythmic genes in LD and ND (5143 genes, deep purple) a diffused circadian pattern of expression was observed, suggesting a displacement or alteration in the daily rhythmic expression of a substantial number of genes due to the extended photoperiod (Figure 9C).

### Module and pathway conservation reveals high level of conservation between *Ostreococcus* and *Chlamydomonas* and a moderate level of conservation between microalgae and *Arabidopsis*

With the aim of determining highly likely functional orthologues between the three different species, information regarding co-expression patterns was integrated with the results obtained previously using the MBBH method. Similar approaches based on the integration of sequence similarity, gene expression profiles and co-expression patterns have been recently successfully applied to determine functional orthologues (Romero-Campero et al., 2013; Das et al., 2016). Thus, it was assumed that two MBBH potential orthologous periodic genes from two different species exhibited a conserved daily pattern when both presented their peak and trough in the same time interval (i.e., same circadian cluster) or when the Pearson correlation coefficient between their expression profiles was higher than 0.98. Therefore, such two genes could be named as expresologues (Das et al., 2016).

According to this criterion approximately 34% of *Arabidopsis* daily rhythmic genes presented an expresologue in *Ostreococcus* or *Chlamydomonas*. This suggests a high level of conservation in spite of the large evolutionary distance between flowering plants and microalgae and presumably, along the plant evolutionary lineage. Interestingly, only the *Arabidopsis* merged cluster of daily rhythmic genes peaking at Dusk was significantly enriched in *Ostreococcus* and *Chlamydomonas* expresologues (*p*–value of 4.84 × 10^−5^ by Fisher's exact test), suggesting that most of these conserved genes maintain a strong influence of light/dark transitions on their expression control. In *Ostreococcus*, 83.62% of the daily rhythmic genes present an expresologue in *Chlamydomonas* but just 36.16% have an expresologue in *Arabidopsis*. In *Chlamydomonas*, 52.71% of the daily rhythmic genes present an expresologue in *Ostreococcus* whereas only 19.05% have an *Arabidopsis* expresologue, which supports a more divergent evolution in this species when compared to *Ostreococcus*.

**Figure 10:**
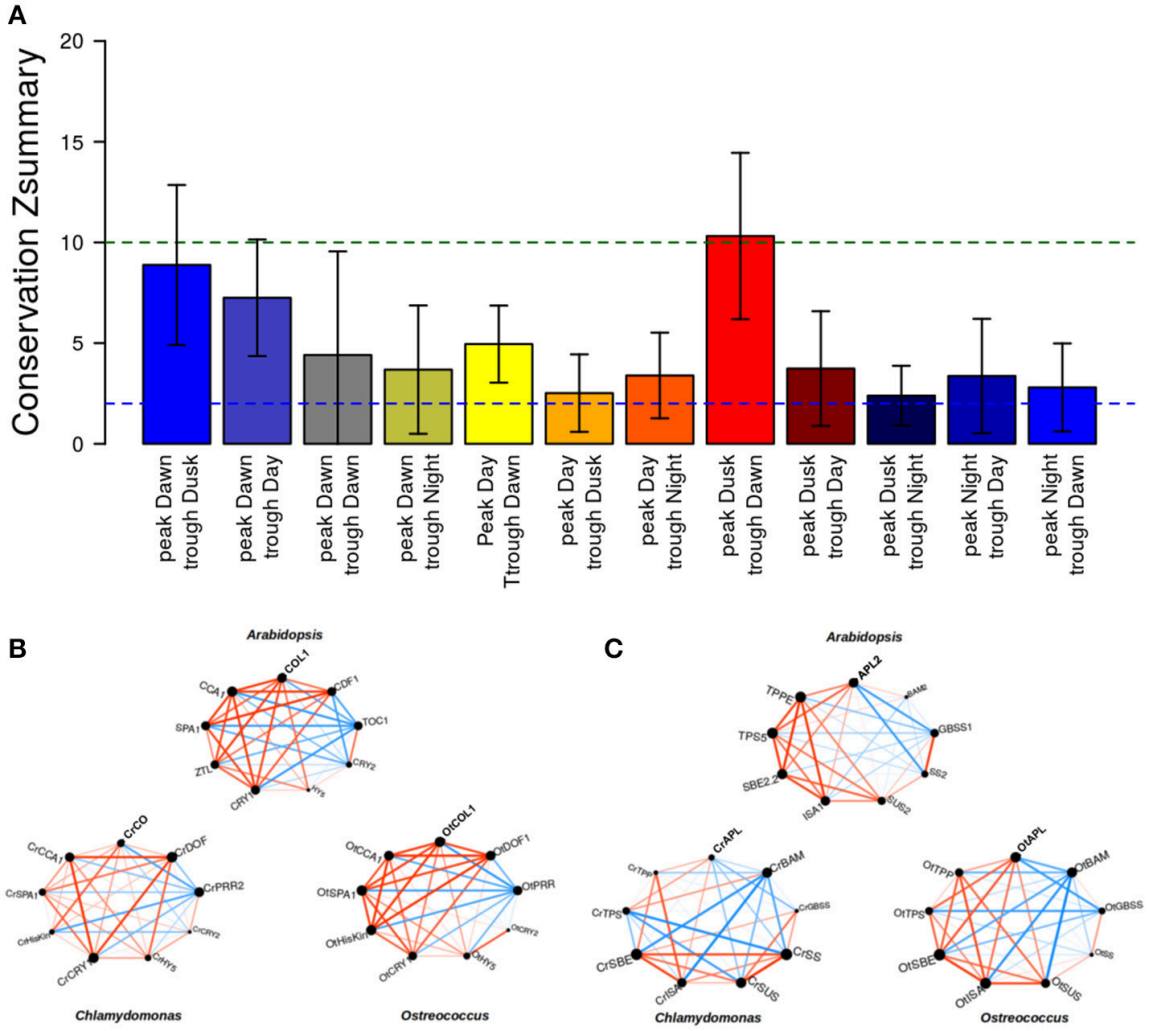
Conservation of circadian patterns in *Arabidopsis, Chlamydomonas*, and *Ostreococcus*. **(A)** The average and standard deviation of the Zsummary conservation score between the three different species was computed for all clusters capturing different daily rhythmic patterns. Only clusters that exhibit a periodic pattern with peak or trough at Dusk or Dawn are significantly conserved over the three different species. **(B)** The co-expression pattern of the key circadian/photoperiod regulators exhibits a high level of conservation in the three species, but it is more evident for *Arabidopsis* and *Ostreococcus*. Red edges represent positive correlation between the corresponding gene expression profiles whereas blue edges indicate negative correlation. Edge width is proportional to the absolute value of the correlation between the corresponding expression profiles. **(C)** The co-expression patterns between key enzymes in starch/sucrose metabolism are highly conserved among the three different species with slight differences in *Chlamydomonas*.

To study the conservation of daily rhythmic patterns in the different clusters among the three species, beyond the comparison between individual gene expression profiles, the Summary Composite Conservation Statistic (Zsummary) was computed as defined in Langfelder et al. (2011) (Figure 10A). A Zsummary value lower than 2 indicates no conservation, a Zsummary value 2–10 implies a moderate conservation, while Zsummary greater than 10 constitutes evidence of a great level of conservation. For each daily rhythmic gene cluster, the Zsummary for the six different possible comparisons (*Arabidopsis* vs. *Ostreococcus, Arabidopsis* vs. *Chlamydomonas, Ostreococcus* vs. *Arabidopsis, Ostreococcus* vs. *Chlamydomonas, Chlamydomonas* vs. *Arabidopsis* and *Chlamydomonas* vs. *Ostreococcus*) was computed and the corresponding average and standard deviation was plotted. In Figure 10A, it can be observed that clusters with a peak at Dawn present a moderate level of daily rhythmic pattern co-expression conservation. In fact, the highest level of conservation was obtained for the cluster with peak at Dawn and trough at Dusk and the cluster peak at Dusk and trough at Dawn (Zsummary > 10). This suggests that evolution has mostly conserved periodic genes with a strong influence from the light/dark and dark/light transitions in their expression profiles, again hinting the importance of these transitory states in plant physiology and their conservation across the entire green lineage.

To corroborate the conservation of co-expression patterns in the three different species, two central pathways identified as daily-regulated in *Arabidopsis* were chosen (Table 2). For these gene sub-clusters, smaller gene co-expression networks were constructed, namely key regulators in circadian/photoperiod system (Figure 10B) and central enzymes in starch/sucrose metabolism (Figure 10C). In these networks, where red edges indicate positive correlations and blue edges indicate negative correlations, a general conservation between the plots can be observed (Figures 10B,C). The circadian/photoperiod plot revealed a high conservation among the three sub-clusters. Nevertheless, a general higher conservation between *Arabidopsis* and *Ostreococcus* display, when compared to *Chlamydomonas* could be observed, indicating again that this microalgae has slightly differentiated from the core *Arabidopsis* model clock (Figure 10B). On the contrary, daily-regulated starch metabolic genes showed a higher conservation degree between *Ostreococcus* and *Chlamydomonas*, maybe revealing that through the course of evolution, additional regulatory steps were incorporated into the higher plant starch synthesis control (Figure 10C). On the whole, this sub-cluster visualization allowed us to compare different subset of genes that had showed a correlation in our general analysis (Supplementary Tables 1–3) further indicating levels of conservation and divergence among the species and constituting efficient tools to initiate the study on the evolution of particular processes.

**Figure 11:**
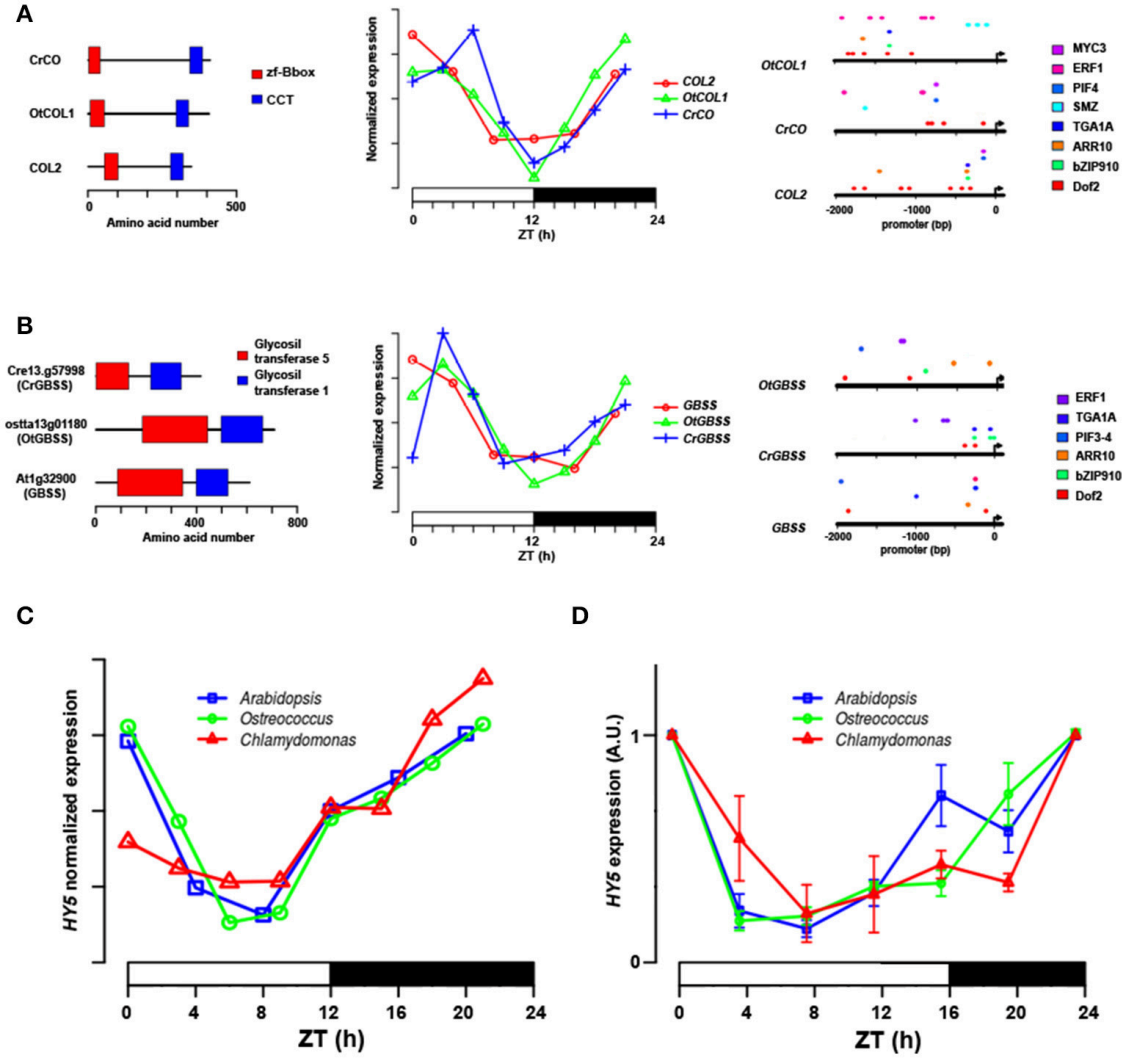
Putative TF orthologues show a conserved protein domain structure, promoter distribution and circadian expression profile in algae and higher plants. **(A)** Gene structure (leftmost), expression in 24 h ND course (middle) and TF binding site in the promoter of *Arabidopsis COL2* gene and putative orthologues from the two microalgae. **(B)** As above, showing *Arabidopsis GBSS* gene and putative orthologues. **(C)** Normalized expression level in 24 h ND photoperiod from the microarray and RNAseq experiments of *HY5* gene from *Arabidopsis* (blue), *CrHY5* from *Chlamydomonas* (red) and *OtHY5* from *Ostreococcus* (green). Notice the high similarity in the expression profile of the three species. **(D)** Expression levels (Arbitrary Units) of *HY5* (blue) from *Arabidopsis, CrHY5* from *Chlamydomonas* (red), and *OtHY5* from *Ostreococcus* (green) in a 24 h course in LD by QPCR. Notice how *HY5* circadian expression pattern is also conserved in the three species, although the daily expression lengthens, strongly supporting the idea that they are true orthologues. Time is shown in hours (h) as Zeitgeber Time (ZT). For each time point of the analysis, three biological replicates and three technical replicates were analyzed. Each point shows standard error bars ± s.e.m.

To further test the veracity of our approach, the promoters of genes showing a high level of conservation both in sequence similarity (Figures 11A,B, leftmost) and co-expression patterns (Figures 11A,B, middle) in the three species were analyzed. As observed in Figures 11A,B rightmost, the promoters of *COLs* (Figure 11A) and *GBSSs* (Figure 11B) orthologues showed also conservation in the TF binding sites identified in the corresponding gene promoters. This result suggests that through evolution, in order to maintain the regulation of these functional networks, whole set of genes have to evolve synchronously and reveal why these networks are so resilient to change even in long evolutionary time scales. Finally, in order to provide an independent validation of the conservation of these daily rhythmic patterns and the capacity to predict orthology in modern plants and algae, the normalized expression of the *Arabidopsis* bZIP gene *HY5* and the putative orthologues detected in this study in *Chlamydomonas* (*CrHY5*) and *Ostreococcus* (*OtHY5*) from the ND microarray analysis (Figure 11C) were plotted and compared with the expression detected in a 24 h LD course by QPCR experiments (Figure 11D). Both plots showed a remarkable similarity, except that the LD expression profiles of HY5 and orthologues prolonged the daily minimal expression levels compared to the ND profile until the last hours of light, when the mRNA levels started to rise again. Therefore, the expression profiles of *HY5* and orthologues showed a very clear conserved pattern, as had been shown in our previous analysis (Figure 8) indicating that the three genes have a high chance of expressing functional orthologues, a line of research that is now being followed in the laboratory.

## Conclusions

As the amount of transcriptomics and phylogenomics data accumulates, the complexity of the evolution of the mechanisms governing the control of gene expression in eukaryotes becomes more evident. A paradigm of a specific pattern that controls the transcriptome occurs under alternating light/dark cycles (Millar, 2016). In photosynthetic organisms this control is particularly important due to their dependence on sunlight for most of their main physiological processes and thus, a complex periodic gene expression mechanism can be found as early as in microalgae (Mittag et al., 2005; Corellou et al., 2009). Here, a Systems Biology approach has been used to dissect this dependence and find out mechanisms anciently controlled by daily rhythms and those that have acquired a new regulation. To achieve this, MBBH tool was developed to help discern orthology from species evolutionarily very distant as algae and modern plants. When used in parallel with gene co-expression networks, a very strong toolset to understand how different processes have conserved a periodic regulation through long evolutionary distances has been built. Furthermore, a web-based tool has been implemented to allow the study of the daily rhythmic evolution of any set of genes from algae to modern plants in a similar way to previous web-based tools that allow researchers to compare diurnal expression profiling between monocots and dicots (Mockler et al., 2007). Together with the available pipelines and databases presented in former papers (Romero-Campero et al., 2013, 2016) they constitute strong resources for the research community.

Using these tools, we confirmed that primitive picoeukaryote microalgae, such as *Ostreococcus*, govern most of its transcriptome (a practical 100%) by a daily rhythmic mechanism under alternating light/dark cycles (Monnier et al., 2010). Other microalgae have reduced this light dependence, but the number of genes regulated by light is still significantly high. This is the case of *Chlamydomonas* (Harris, 2001), which can perform some complex physiological processes independently of light/dark cycles, now controlled by other factors such as external biotic stimuli. Following a similar tendency, *Arabidopsis* has reduced the temporal dependence of its gene expression control under light/dark cycles to just over a third of its transcriptome (Michael et al., 2008) but is still significantly higher than the 10–15% of genes showing a periodic control in mammals (Lowrey and Takahashi, 2011). Nevertheless, it has been reported that up to 89% of the *Arabidopsis* transcriptome could follow a daily rhythmic pattern when different environmental signals, such as temperature, are combined with light/dark cycles (Michael et al., 2008). Therefore, plants seem to possess several molecular mechanisms to integrate diverse environmental signals beside light/dark cycles to exert daily rhythmic regulation over their transcriptome that are missing in microalgae. Temperature seems to be a key factor among these signals. The lack of potential microalgal orthologues for key genes in the evening loop such as GI and ZTL, that exert the temperature compensation to the clock in *Arabidopsis*, could explain these differences.

Finally, the clustering analysis performed attending to gene rhythmic expression has shown that the co-expression networks in the three organisms are distributed in the same order as the clock, giving the circadian timer a real associative and temporal dimension that participates in the coordination of different physiological processes such as ribosome synthesis or starch metabolism. In algae, those clusters that showed maximum expression during the light/dark transitions are the most abundant, while in *Arabidopsis*, these clusters containing orthologous genes, seem to be expressed during the dark or light periods. It seems then that *Arabidopsis* has been able to predict dawn and dusk in a better way than algae and has advanced gene expression in order to anticipate these transitions. Moreover, when a global conservation analysis is performed, only genes with their peaks and troughs at dawn and dusk are strongly conserved between microalgae and plants, again suggesting a diversification in the temporal control of gene expression during their evolution. Finally, we have also shown the powerful predictable capacity of the approach by picking up putative orthologues that may result in good candidates for further studies in the future. This approach could help to better understand daily rhythmic regulation in algal systems, which may be important to understand the more complex mechanisms of higher plants and shade some light on how these mechanisms have evolved in the green lineage.

## Author contributions

Pd and FR performed bioinformatics analysis. Pd and MR carried out experimental work. FV, JR, and FR conceived the study, interpreted the results and wrote the paper. All authors read and approved the manuscript.

### Conflict of interest statement

The authors declare that the research was conducted in the absence of any commercial or financial relationships that could be construed as a potential conflict of interest.

## References

[B1] Adams S. Carré I. A. (2011). Downstream of the plant circadian clock: output pathways for the control of physiology and development. Essays Biochem. 49, 53–69. 10.1042/bse0490053 21819384

[B2] Alexa A. Rahnenfuhrer J. (2016). TopGO: Enrichment Analysis for Gene Ontology. R package version 2.26.20.

[B3] Aoki K. Ogata Y. Shibata D. (2007). Approaches for extracting practical information from gene coexpression networks in plant biology. Plant Cell Physiol. 48, 381–390. 10.1093/pcp/pcm013 17251202

[B4] Aucoin H. R. Gardner J. Boyle N. R. (2016). Omics in Chlamydomonas for biofuel production. Subcell. Biochem. 86, 447–469. 10.1007/978-3-319-25979-6_18 27023246

[B5] Barabási A. L. (2015). Network Science. Cambridge, MA: Cambridge University Press.

[B6] Barrett T. Wilhite S. E. Ledoux P. Evangelista C. Kim I. F. Tomashevsky M.. (2013). NCBI GEO: archive for functional genomics data sets—update. Nucleic Acids Res. 41, D991–D995. 10.1093/nar/gks1193 23193258 PMC3531084

[B7] Blanc-Mathieu R. Verhelst B. Derelle E. Rombauts S. Bouget F.-Y. Carré I.. (2014). An improved genome of the model marine alga *Ostreococcus tauri* unfolds by assessing Illumina *de novo* assemblies. BMC Genomics 15:1103. 10.1186/1471-2164-15-1103 25494611 PMC4378021

[B8] Bläsing O. E. Gibon Y. Günther M. Höhne M. Morcuende R. Osuna D.. (2005). Sugars and circadian regulation make major contributions to the global regulation of diurnal gene expression in *Arabidopsis*. Plant Cell 17, 3257–3281. 10.1105/tpc.105.035261 16299223 PMC1315368

[B9] Corellou F. Schwartz C. Motta J. P. Djouani-Tahri E. B. Sanchez F. Bouget F. Y. (2009). Clocks in the green lineage: comparative functional analysis of the circadian architecture of the picoeukaryote *Ostreococcus*. Plant Cell 21, 3436–3449. 10.1105/tpc.109.068825 19948792 PMC2798331

[B10] Covington M. F. Maloof J. N. Straume M. Kay S. A. Harmer S. L. (2008). Global transcriptome analysis reveals circadian regulation of key pathways in plant growth and development. Genome Biol. 9:130. 10.1186/gb-2008-9-8-r130 18710561 PMC2575520

[B11] Csárdi G. Nepusz T. (2006). The Igraph Software Package for Complex Network Research. Inter Journal Complex Systems (1695).

[B12] Dalquen D. A. Dessimoz C. (2013). Bidirectional best hits miss many orthologs in duplication-rich clades such as plants and animals. Genome Biol. Evol. 5, 1800–1806. 10.1093/gbe/evt132 24013106 PMC3814191

[B13] Das M. Haberer G. Panda A. Das Laha S. Ghosh T. C. Schäffner A. R. (2016). Expression pattern similarities support the prediction of orthologs retaining common functions after gene duplication events. Plant Physiol. 171, 2343–2357. 10.1104/pp.15.01207 27303025 PMC4972257

[B14] de Vries J. Stanton A. Archibald J. M. Gould S. B. (2016). Streptophyte terrestralization in light of plastid evolution. Trends Plant Sci. 21, 467–476. 10.1016/j.tplants.2016.01.021 26895731

[B15] Delwiche C. F. Cooper E. D. (2015). The evolutionary origin of a terrestrial flora. Curr. Biol. 25, 899–910. 10.1016/j.cub.2015.08.029 26439353

[B16] Deng X. Fan X. Li P. Fei X. (2015). A photoperiod-regulating gene CONSTANS is correlated to lipid biosynthesis in *Chlamydomonas reinhardtii*. Biomed. Res. Int. 2015:715020. 10.1155/2015/715020 25654119 PMC4310486

[B17] Derelle E. Ferraz C. Rombauts S. Rouzé P. Worden A. Z. Robbens S.. (2006). Genome analysis of the smallest free-living eukaryote *Ostreococcus tauri* unveils many unique features. Proc. Natl. Acad. Sci. U.S.A. 103, 11647–11652. 10.1073/pnas.0604795103 16868079 PMC1544224

[B18] Dessimoz C. Gabaldon T. Roos D. S. Sonnhammer E. L. L. Herrero J. (2012). Towards community standards in the quest for orthologs. Bioinformatics 28, 900–904. 10.1093/bioinformatics/bts050 22332236 PMC3307119

[B19] Djouani-Tahri E. B. Christie J. M. Sanchez-Ferandin S. Sanchez F. Bouget F.-Y. Corellou F. (2011). A eukaryotic LOV-histidine kinase with circadian clock function in the picoalga *Ostreococcus*. Plant J. 65, 578–588. 10.1111/j.1365-313X.2010.04444.x 21235644

[B20] Fornara F. Panigrahi K. C. S. Gissot L. Sauerbrunn N. Rühl M. Jarillo J. A.. (2009). Arabidopsis DOF transcription factors act redundantly to reduce CONSTANS expression and are essential for a photoperiodic flowering response. Dev. Cell 17, 75–86. 10.1016/j.devcel.2009.06.015 19619493

[B21] Gautier L. Cope L. Bolstad B. M. Irizarry R. A. (2004). affy—analysis of Affymetrix GeneChip data at the probe level. Bioinformatics 20, 307–315. 10.1093/bioinformatics/btg405 14960456

[B22] Gehan M. A. Greenham K. Mockler T. C. McClung C. R. (2015). Transcriptional networks-crops, clocks and abiotic stress. Curr. Opin. Plant Biol. 24, 39–46. 10.1016/j.pbi.2015.01.004 25646668

[B23] Goodstein D. M. Shu S. Howson R. Neupane R. Hayes R. D. Fazo J.. (2012). Phytozome: a comparative platform for green plan genomics. Nucleic Acids Res. 40, D1178–D1186. 10.1093/nar/gkr944 22110026 PMC3245001

[B24] Harris E. H. (2001). Chlamydomonas as a model organism. Annu. Rev. Plant Physiol. Plant Mol. Biol. 52, 363–406. 10.1146/annurev.arplant.52.1.363 11337403

[B25] Henderson G. P. Gan L. Jensen G. J. (2007). 3-D ultrastructure of *O. tauri*: electron cryotomography of an entire eukaryotic cell. PLoS ONE 2:e749. 10.1371/journal.pone.0000749 17710148 PMC1939878

[B26] Huang W. Pérez-García P. Pokhilko A. Millar A. J. Antoshechkin I. Riechmann J. L.. (2012). Mapping the core of the *Arabidopsis* circadian clock defines the network structure of the oscillator. Science 336, 75–79. 10.1126/science.1219075 22403178

[B27] Imaizumi T. Schultz T. F. Harmon F. G. Ho L. A. Kay S. A. (2005). FKF1 F-box protein mediates cyclic degradation of a repressor of CONSTANS in *Arabidopsis*. Science 309, 293–297. 10.1126/science.1110586 16002617

[B28] Jin J. P. Tian F. Yang D. C. Meng Y. Q. Kong L. Luo J. C.. (2017). PlantTFDB 4.0: toward a central hub for transcription factors and regulatory interactions in plants. Nucleic Acids Res. 45, 1040–1045. 10.1093/nar/gkw982 27924042 PMC5210657

[B29] Kamioka M. Takao S. Suzuki T. Taki K. Higashiyama T. Kinoshita T.. (2016). Direct repression of evening genes by CIRCADIAN CLOCK-ASSOCIATED 1 in the *Arabidopsis* circadian clock. Plant Cell 28, 696–711. 10.1105/tpc.15.00737 26941090 PMC4826007

[B30] Keller M. D. Selvin R. C. Claus W. Guillard R. R. L. (1987). Media for the culture of marine ultraphytoplankton. J. Phycol. 23, 633–638. 10.1111/j.1529-8817.1987.tb04217.x

[B31] Kloosterman B. Abelenda J. A. Gomez M. D. M. C. Oortwijn M. de Boer J. M. Kowitwanich K. (2013). Naturally occurring allele diversity allows potato cultivation in northern latitudes. Nature 495, 246–250. 10.1038/nature11912 23467094

[B32] Koornneef M. Meinke D. (2010). The development of *Arabidopsis* as a model plant. Plant J. 61, 909–921. 10.1111/j.1365-313X.2009.04086.x 20409266

[B33] Langfelder P. Horvath S. (2012). Fast R functions for robust correlations and hierarchical clustering. J. Stat. Softw. 46, 1–17. 10.18637/jss.v046.i11 23050260 PMC3465711

[B34] Langfelder P. Luo R. Oldham M. C. Horvath S. (2011). Is my network module preserved and reproducible? PLoS Comput. Biol. 7:e1001057. 10.1371/journal.pcbi.1001057 21283776 PMC3024255

[B35] Liu T. Carlsson J. Takeuchi T. Newton L. Farré E. M. (2013). Direct regulation of abiotic responses by the *Arabidopsis* circadian clock component PRR7. Plant J. 76, 101–114. 10.1111/tpj.12276 23808423

[B36] Liu T. Newton L. Liu M. J. Shiu S. H. Farré E. M. (2016). A G-Box-like motif is necessary for transcriptional regulation by circadian pseudo-response regulators in *Arabidopsis*. Plant Physiol. 170, 528–539. 10.1104/pp.15.01562 26586835 PMC4704597

[B37] Lowrey P. L. Takahashi J. S. (2011). Genetics of circadian rhythms in mammalian model organisms. Adv. Genet. 74, 175–230. 10.1016/b978-0-12-387690-4.00006-4 21924978 PMC3709251

[B38] Lucas-Reina E. Romero-Campero F. J. Romero J. M. Valverde F. (2015). An evolutionarily conserved DOF-CONSTANS module controls plant photoperiodic signaling. Plant Physiol. 168, 561–574. 10.1104/pp.15.00321 25897001 PMC4453789

[B39] Merchant S. S. Prochnik S. E. Vallon O. Harris E. H. Karpowicz S. J. Witman G. B.. (2007). The Chlamydomonas genome reveals the evolution of key animal and plant functions. Science 318, 245–250. 10.1126/science.1143609 17932292 PMC2875087

[B40] Michael T. P. Mockler T. C. Breton G. McEntee C. Byer A. Trout J. D.. (2008). Network discovery pipeline elucidates conserved time-of-day-specific cis-regulatory modules. PLoS Genet. 4:e14. 10.1371/journal.pgen.0040014 18248097 PMC2222925

[B41] Millar A. J. (2016). The intracellular dynamics of circadian clocks reach for the light of ecology and evolution. Annu. Rev. Plant Biol. 67, 595–618. 10.1146/annurev-arplant-043014-115619 26653934

[B42] Mittag M. Kiaulehn S. Johnson C. H. (2005). The circadian clock in *Chlamydomonas reinhardtii*. What is it for? What is it similar to? Plant Physiol. 137, 399–409. 10.1104/pp.104.052415 15710681 PMC1065344

[B43] Miyazaki Y. Takase T. Kiyosue T. (2015). ZEITLUPE positively regulates hypocotyl elongation at warm temperature under light in *Arabidopsis thaliana*. Plant Signal. Behav. 10:e998540. 10.1080/15592324.2014.998540 26039487 PMC4623253

[B44] Mizuno T. Nakamichi N. (2005). Pseudo-Response Regulators (PRRs) or True Oscillator Components (TOCs). Plant Cell Physiol. 46, 677–685. 10.1093/pcp/pci087 15767264

[B45] Mockler T. C. Michael T. P. Priest H. D. Shen R. Sullivan C. M. Givan S. A.. (2007). The DIURNAL project: DIURNAL and circadian expression profiling, model-based pattern matching, and promoter analysis. Cold Spring Harb. Symp. Quant. Biol. 72, 353–363. 10.1101/sqb.2007.72.006 18419293

[B46] Monnier A. Liverani S. Bouvet R. Jesson B. Smith J. Q. Mosser J.. (2010). Orchestrated transcription of biological processes in the marine picoeukaryote *Ostreococcus* exposed to light/dark cycles. BMC Genomics 22:192. 10.1186/1471-2164-11-192 20307298 PMC2850359

[B47] Mora-García S. de Leone M. J. Yanovsky M. (2017). Time to grow: circadian regulation of growth and metabolism in photosynthetic organisms. Curr. Opin. Plant Biol. 35, 84–90. 10.1016/j.pbi.2016.11.009 27912128

[B48] Nakamichi N. Kiba T. Kamioka M. Suzuki T. Yamashino T. Higashiyama T.. (2012). Transcriptional repressor PRR5 directly regulates clock-output pathways. Proc. Natl. Acad. Sci. U.S.A. 109, 17123–17128. 10.1073/pnas.1205156109 23027938 PMC3479524

[B49] Nohales M. A. Kay S. A. (2016). Molecular mechanisms at the core of the plant circadian oscillator. Nat. Struct. Mol. Biol. 23, 1061–1069. 10.1038/nsmb.3327 27922614 PMC7750160

[B50] Ortiz-Marchena M. I. Albi T. Lucas-Reina E. Said F. E. Romero-Campero F. J. Cano B.. (2014). Photoperiodic control of carbon distribution during the floral transition in *Arabidopsis*. Plant Cell 26, 565–584. 10.1105/tpc.114.122721 24563199 PMC3967026

[B51] Pagès H. Aboyoun P. Gentleman R. DebRoy S. (2016). Biostrings: String Objects Representing Biological Sequences, and Matching Algorithms. R package version 2.42.41.

[B52] Palenik B. Grimwood J. Aerts A. Rouzé P. Salamov A. Putnam N.. (2007). The tiny eukaryote *Ostreococcus* provides genomic insights into the paradox of plankton speciation. Proc. Natl. Acad. Sci. U.S.A. 104, 7705–7710. 10.1073/pnas.0611046104 17460045 PMC1863510

[B53] Punta M. Coggill P. Eberhardt R. Mistry J. Tate J. Boursnell C.. (2012). The Pfam protein families database. Nucleid Acids Res. 40, 290–301. 10.1093/nar/gkr1065 22127870 PMC3245129

[B54] Raven J. A. Beardall J. Larkum A. W. D. Sánchez-Baracaldo P. (2013). Interactions of photosynthesis with genome size and function. Philos. Trans. R. Soc. Lond. B Biol. Sci. 368:20120264. 10.1098/rstb.2012.0264 23754816 PMC3685465

[B55] Rensing S. A. (2014). Gene duplication as a driver of plant morphogenetic evolution. Curr. Opin. Plant Biol. 17, 43–48. 10.1016/j.pbi.2013.11.002 24507493

[B56] Romero-Campero F. J. Lucas-Reina E. Said F. E. Romero J. M. Valverde F. (2013). A contribution to the study of plant development evolution based on gene co-expression networks. Front. Plant Sci. 4:291. 10.3389/fpls.2013.00291 23935602 PMC3732916

[B57] Romero-Campero F. J. Perez-Hurtado I. Lucas-Reina E. Romero J. M. Valverde F. (2016). ChlamyNET: a Chlamydomonas gene co-expression network reveals global properties of the transcriptome and the early setup of key co-expression patterns in the green lineage. BMC Genomics 17:227. 10.1186/s12864-016-2564-y 26968660 PMC4788957

[B58] Rugnone M. L. Faigón-Soverna A. Sanchez S. E. Schlaen R. G. Hernando C. E. Seymour D. K.. (2013). LNK genes integrate light and clock signaling networks at the core of the *Arabidopsis* oscillator. Proc. Natl. Acad. Sci. U.S.A. 110, 12120–12125. 10.1073/pnas.1302170110 23818596 PMC3718124

[B59] Ruprecht C. Proost S. Hernandez-Coronado M. Ortiz-Ramirez C. Lang D. Rensing S. A.. (2017). Phylogenomic analysis of gene co-expression networks reveals the evolution of functional modules. Plant J. 90, 447–465. 10.1111/tpj.13502 28161902

[B60] Satbhai S. B. Yamashino T. Okada R. Nomoto Y. Mizuno T. Tezuka Y.. (2011). Pseudo-response regulator (PRR) homologues of the moss *Physcomitrella patens*: insights into the evolution of the PRR family in land plants. DNA Res. 18, 39–52. 10.1093/dnares/dsq033 21186242 PMC3041508

[B61] Schreiber K. J. Bentham A. Williams S. J. Kobe B. Staskawicz B. J. (2016). Multiple domain associations within the *Arabidopsis* immune receptor RPP1 regulate the activation of programmed cell death. PLOS Pathog. 12:e1005769. 10.1371/journal.ppat.1005769 27427964 PMC4948778

[B62] Serrano G. Herrera-Palau R. Romero J. M. Serrano A. Coupland G. Valverde F. (2009). Chlamydomonas CONSTANS and the evolution of plant photoperiodic signaling. Curr. Biol. 19, 359–368. 10.1016/j.cub.2009.01.044 19230666

[B63] Serrano-Bueno G. Romero-Campero F. J. Lucas-Reina E. I. Romero J. M. Valverde F. (2017). Evolution of photoperiod sensing in plants and algae. Curr. Opin. Plant Biol. 37, 10–17. 10.1016/j.pbi.2017.03.007 28391047

[B64] Shannon P. Markiel A. Ozier O. Baliga N. S. Wang J. T. Ramage D.. (2003). Cytoscape: a software environment for integrated models of biomolecular interaction networks. Genome Res. 13, 2498–2504. 10.1101/gr.1239303 14597658 PMC403769

[B65] Sorokina O. Corellou F. Dauvillée D. Sorokin A. Goryanin I. Ball S.. (2011). Microarray data can predict diurnal changes of starch content in the picoalga *Ostreococcus*. BMC Systems Biol. 5:36. 10.1186/1752-0509-5-36 21352558 PMC3056741

[B66] Sterck L. Billiau K. Abeel T. Rouzé P. Van der Peer Y. (2012). ORCAE: online resource for community annotation of eukaryotes. Nat. Methods 9:1041. 10.1038/nmeth.2242 23132114

[B67] Supek F. Bošnjak M. Škunca N. Šmuc T. (2011). REVIGO summarizes and visualizes long lists of gene ontology terms. PLoS ONE 6:e21800. 10.1371/journal.pone.0021800 21789182 PMC3138752

[B68] Thaben P. F. Westermark P. O. (2014). Detecting rhythms in time series with RAIN. J. Biol. Rhythms 29, 391–400. 10.1177/0748730414553029 25326247 PMC4266694

[B69] Thommen Q. Pfeuty B. Schatt P. Bijoux A. Bouget F. Y. Lefranc M. (2015). Probing entrainment of *Ostreococcus tauri* circadian clock by green and blue light through a mathematical modeling approach. Front. Genet. 6:65. 10.3389/fgene.2015.00065 25774167 PMC4343026

[B70] Trachana K. Jensen L. J. Bork P. (2010). Evolution and regulation of cellular periodic processes: a role for paralogues. EMBO Rep. 11, 233–238. 10.1038/embor.2010.9 20168326 PMC2838706

[B71] Trapnell C. Roberts A. Goff L. Pertea G. Kim D. Kelley D. R.. (2012). Differential gene and transcript expression analysis of RNA-seq experiments with TopHat and Cufflinks. Nat. Protoc. 7, 562–578. 10.1038/nprot.2012.016 22383036 PMC3334321

[B72] Valverde F. (2011). CONSTANS and the evolutionary origin of photoperiodic timing of flowering. J. Exp. Bot. 62, 2453–2463. 10.1093/jxb/erq449 21239381

[B73] Van Norman J. M. Benfey P. N. (2009). *Arabidopsis thaliana* as a model organism in systems biology. Wiley Interdiscip. Rev. Syst. Biol. Med. 1, 372–379. 10.1002/wsbm.25 20228888 PMC2836806

[B74] Wang M. Yuan D. Gao W. Li Y. Tan J. Zhang X. (2013). A comparative genome analysis of PME and PMEI families reveals the evolution of pectin metabolism in plant cell walls. PLoS ONE 8:e72082. 10.1371/journal.pone.0072082 23951288 PMC3741192

[B75] Wolf Y. I. Koonin E. V. (2012). A tight link between orthologs and bidirectional best hits in bacterial and archaeal genomes. Genome Biol. Evol. 4, 1286–1294. 10.1093/gbe/evs100 23160176 PMC3542571

[B76] Yu G. Wang L. Han Y. He Q. (2012). clusterProfiler: an R package for comparing biological themes among gene clusters. OMICS 16, 284–287. 10.1089/omi.2011.0118 22455463 PMC3339379

[B77] Zhang J. D. Biczok R. Ruschhaupt M. (2015). ddCt: The ddCt Algorithm for the Analysis of Quantitative Real-Time PCR (qRT-PCR). R package version 1.30.30.

[B78] Zhu T. Nevo E. Sun D. Peng J. (2012). Phylogenetic analyses unravel the evolutionary history of NAC proteins in plants. Evolution 66, 1833–1848. 10.1111/j.1558-5646.2011.01553.x 22671550

[B79] Zones J. M. Blaby I. K. Merchant S. S. Umen J. G. (2015). High-resolution profiling of a synchronized diurnal transcriptome from *Chlamydomonas reinhardtii* reveals continuous cell and metabolic differentiation. Plant Cell 27, 2743–2769. 10.1105/tpc.15.00498 26432862 PMC4682324

